# LDD-YOLOv11: Lightweight Defect Detection YOLOv11 Network for Automatic Surface Crack Identification

**DOI:** 10.3390/s26216781

**Published:** 2026-10-26

**Authors:** Youzheng Wang, Wenyu Li, Shuyi Fan, Fuwei Zhang, Zhenxin Diao, Siyuan Liu, Haining Jia, Qizhen Zhou

**Affiliations:** 1 State Key Laboratory of Disaster Prevention and Mitigation of Explosion and Impact, Army Engineering University of People’s Liberation Army of China, Nanjing 210007, China; wangyouzheng0226@163.com (Y.W.); lwy19120729973@163.com (W.L.); 19318971804@163.com (S.F.); 17735066169@163.com (F.Z.); 19301165429@163.com (Z.D.); 15005169961@163.com (H.J.); zhouqz@aeu.edu.cn (Q.Z.); 2 College of Defense Engineering, Army Engineering University of People’s Liberation Army of China, Nanjing 210007, China

**Keywords:** crack detection, YOLOv11, unmanned platforms, computer vision, knowledge distillation

## Abstract

Early detection of fine cracks is crucial for preventing structural failures in civil engineering. Conventional manual inspection approaches for structural surface cracks often suffer from limited operational efficiency and inevitable subjective bias, as visual assessment heavily relies on inspectors’ personal experience, fatigue levels, and on-site lighting conditions. In contrast, deep-learning-based crack detection methods have become mainstream alternatives to manual inspection. However, these approaches encounter a host of practical challenges when deployed on unmanned inspection platforms. Complex background interference limits the accurate extraction of fine-grained crack features; furthermore, insufficiently annotated datasets and cumbersome network architectures that fail to support lightweight real-time inference jointly degrade model generalization in diverse field operating environments. To address these issues, this paper proposes a Lightweight Defect Detection YOLOv11 Network (LDD-YOLOv11), a crack detection algorithm that combines directional multi-scale feature enhancement with a teacher–student knowledge distillation mechanism. The technological innovations of this research are twofold. On the one hand, LDD-YOLOv11 embeds two dedicated feature enhancement modules, namely the Light-Material Aware Adapter (LMA) and the Direction-Material Constrained Focus (DMC-Focus) module, into the original YOLOv11 backbone and neck to strengthen multi-scale fine crack feature representation. On the other hand, we adopt a tailored Dual Perception Focusing Distillation (DPFD) strategy to boost feature-level knowledge transfer and improve overall generalization robustness. Evaluated on a self-constructed urban building crack dataset, the proposed model achieves a mean average precision at 0.5 (mAP@0.5) of 90.50% with only 3.20 M parameters and an inference speed of 67.7 FPS. Compared with the fully trained YOLOv11n baseline (88.30% mAP@0.5), mAP@0.5 is increased by 2.2 percentage points while maintaining favorable lightweight characteristics. Furthermore, its accuracy and efficiency surpass those of mainstream crack detection models such as U-Net and DeepCrack. Cross-dataset generalization experiments and edge deployment simulation further validate the model’s adaptability to complex scenarios and practical deployment potential. Consequently, LDD-YOLOv11 proves effective for automatic structural crack inspection and demonstrates significant engineering application value.

## 1. Introduction

With the rapid advancement of urbanization and the continuous aging of infrastructure, core facilities including buildings, pavements, and underground garages sustain long-term heavy loads and are subject to persistent exposure to adverse environments. Consequently, such structures are susceptible to structural defects like surface cracks, concrete spalling, and pavement potholes, which severely compromise their operational safety and long-term service performance [1]. Among various structural diseases, cracks are the most representative early damage signals. They not only directly reflect the internal stress state of the structure but may also gradually cause a series of performance degradation problems such as steel bar corrosion and concrete spalling. Therefore, precise detection of early fine cracks is a key step in scientifically assessing the durability of engineering structures such as buildings and roads and effectively preventing potential deterioration risks.

Traditional crack detection mostly relies on manual visual inspection [2]. Qualified inspectors need to carry out on-site monitoring and assessment. This process is time-consuming and laborious, with many human interference factors, and is prone to misjudgment and missed judgment problems [3]. Existing research indicates that approximately 50% of building condition assessment results are incorrect or significantly influenced by the experience of inspection personnel [4], making it difficult to achieve large-scale and standardized condition assessment.

In recent years, deep-learning-based crack detection methods have gradually replaced manual inspection as mainstream schemes [5], yet they still face some core practical bottlenecks restricting deployment on unmanned inspection platforms. First, conventional networks lack targeted feature enhancement modules, resulting in weak crack feature extraction under uneven illumination and complex backgrounds [6], with insufficient adaptability to diverse on-site building scenes. Second, most mainstream detection architectures adopt bulky structures with heavy computational overhead, failing to support lightweight low-latency reasoning on edge devices with tight computing resource constraints [7,8]. Third, real-world annotated crack datasets are generally scarce, and common training strategies lack targeted knowledge optimization, leading to unsatisfactory detection precision and weak cross-scene generalization ability.

From the technical paradigm perspective, existing vision-based crack detection approaches can be generally divided into two categories. The first category is physics-guided multi-modal detection methods, which integrate specialized sensing signals such as terahertz time-domain spectroscopy and micro-X-ray fluorescence and embed physical prior knowledge into network optimization [9]. Such methods achieve high accuracy for material-level internal defects but depend on dedicated sensing hardware and strictly controlled acquisition conditions, making them difficult to adapt to low-cost mobile inspection scenarios. The second category is visible-light RGB vision methods, which only use RGB images captured by ordinary cameras and extract crack visual features through deep learning models. Benefiting from low deployment cost and high environmental adaptability, RGB-based detection has become the mainstream technical route for unmanned mobile structural inspection. Recent studies have focused on lightweight network design and edge deployment optimization to enable real-time inference on resource-constrained platforms: Lite-V2 achieves 95.7% classification accuracy with only 0.28 M parameters and verifies inference performance on Raspberry Pi [10]; ZoomHead optimizes the detection head structure to balance accuracy and speed for slender crack detection on edge devices [11]. However, most lightweight crack detectors still suffer from insufficient feature capture ability for ultra-fine cracks and lack systematic verification for real-world inspection scenarios.

This work falls into the visible-light RGB vision paradigm. Targeting the above pain points, this study proposes LDD-YOLOv11, a lightweight fine crack detection algorithm for building surfaces deployed on edge unmanned platforms. The proposed method is developed purely based on visible-light RGB images from ordinary inspection cameras and does not introduce multi-modal physical sensing, physical prior constraints, or material mechanics models. It should be clarified that the term “Material” in the names of the proposed Light-Material Aware Adapter (LMA) and Direction-Material Constrained Focus (DMC-Focus) module refers exclusively to the apparent texture and grayscale distribution features of building surfaces in RGB images rather than physical material properties or multi-modal physical sensing. All the proposed modules are pure visual processing units, and no physics-guided mechanisms or physical signal processing is involved in this work. First, we adopt the lightweight YOLOv11n backbone to control computational overhead and support real-time inference on edge hardware. Second, we embed a customized attention module to strengthen weak crack visual feature extraction and suppress complex background noise. Third, we introduce the DPFD dual-perception knowledge distillation module to transfer high-value crack feature knowledge from the teacher model, which mitigates performance degradation caused by insufficient training data and elevates cross-scene generalization. Finally, the loss function is refined to enhance fitting accuracy for blurry, low-contrast tiny cracks.

In summary, the main contributions of this work are as follows:

1. **Lightweight Synergistic Detection Architecture**: A lightweight crack detection framework named LDD-YOLOv11 is proposed, which integrates a Light-Material Aware Adapter (LMA), a Direction-Material Constrained Focus (DMC-Focus) module, and a Dual Perception Focused Distillation (DPFD) strategy into the YOLOv11 backbone. The nonlinear synergistic gain among modules is verified, achieving improved detection accuracy with only 3.2 M parameters.
2. **Dual Perception Focused Distillation Strategy**: The DPFD strategy transfers knowledge from the teacher model at the feature level, response level, and prototype level, with adaptive weight balancing. It effectively improves the generalization ability of the lightweight model for weak crack features under complex illumination and background conditions.
3. **Real-Scene Dataset with Building-Level Splitting**: A building crack dataset for real inspection scenarios is constructed. A building-level data splitting strategy is adopted to eliminate data leakage caused by random image-level splitting, and complete statistical attributes of crack geometry and imaging conditions are supplemented to improve the credibility and engineering reference value of experiments.
4. **Edge Deployment Feasibility Verification**: Systematic computational complexity analysis and edge deployment evaluation are carried out. Through quantitative comparison of parameters, GFLOPs, CPU inference performance, and theoretical edge performance estimation, the deployment potential of the model on low-power edge inspection platforms is comprehensively verified.

The remaining structure of this article is arranged as follows: Section 2 summarizes the current research status and core challenges in the field of crack detection based on deep learning and unmanned platforms; Section 3 presents the overall detailed architecture of YOLOv11 and LDD-YOLOv11 and specifically explains the application modules of each part of the proposed algorithm; Section 4 focuses on the preparation of the building crack dataset used in the experiment, the implementation of the experiment, and the analysis of the results. Section 5 begins the discussion, points out the shortcomings of current research, and explores future research directions; Section 6 summarizes the full text, summarizing the research achievements and related contributions of this study.

## 2. Literature Review

### 2.1. Data-Driven Crack Recognition Methods

Before the popularization of intelligent detection technology, building crack inspection primarily relied on traditional manual detection and equipment-assisted detection.

Manual inspection adopts visual observation and simple measuring tools, which are low-cost and easy to operate but only suitable for small-scale and local crack detection [12,13]. Traditional manual detection is limited by low efficiency, strong subjectivity, and poor operational safety. These drawbacks restrict its application in large-scale and complex inspection scenarios and fail to support accurate quantitative analysis of crack parameters.

As an important supplement to manual inspection, auxiliary detection techniques, including ultrasonic testing [14], improve detection performance by capturing structural physical characteristics. Nevertheless, such methods suffer from poor equipment portability, high inspection costs, and susceptibility to environmental interference, making them unsuitable for large-scale and efficient crack inspection tasks.

Early automatic crack recognition methods are dominated by geometric algorithms and traditional machine learning approaches [5]. These methods rely on manually designed edge and texture features, lacking robustness against blurred images and uneven illumination, which are widely encountered in actual engineering scenarios [6]. Meanwhile, to realize efficient, continuous on-site monitoring, researchers have turned to deploying detection algorithms on unmanned inspection platforms. Unfortunately, handcrafted features extracted by traditional algorithms cannot adapt to complex field images captured by such devices, resulting in highly unstable crack detection accuracy [15].

To overcome the limitations of manual engineering, deep learning models have become the mainstream solution for unmanned crack inspection, as they automatically learn discriminative crack features from labeled datasets [16]. Current deep learning crack detection approaches fall into three mainstream branches: CNN-based classification, object detection localization, and pixel-level semantic segmentation. Each category adopts distinct algorithmic pipelines to implement crack detection and quantitative analysis.

The CNN-based paradigm frames crack detection as an image classification task, which identifies crack-containing images via transfer learning on pre-trained backbones such as ResNet [17] and VGGNet [18]. Golding et al. [19] validated that grayscale CNN models achieve comparable performance to RGB counterparts. Li and Zhao [20] proposed a CNN architecture reaching 99.39% detection accuracy, demonstrating the network’s efficient computation. Object detection methods rely on Faster R-CNN [21], SSD [22], YOLO [23], and other frameworks to output crack bounding boxes. Zhu et al. [24] proved that SSD-MobileNetv2 balances detection precision and compact parameter volume to fit lightweight hardware requirements. Semantic segmentation models, including U-Net [25] and FCN [26], deliver pixel-level crack segmentation. Kim et al. [27] presented GTAU-Net, integrating a gradient transformer and self-attention with 99.42% detection accuracy. Zhang et al. [28] combined attention modules with FCN for asphalt pavement crack segmentation, enabling precise pixel-wise identification and adapting to unmanned vehicle inspection with balanced accuracy and latency.

Despite these advances, existing deep learning methods still suffer from inherent deficiencies that restrict deployment on edge unmanned devices [7]. Firstly, slender, low-contrast blurry cracks are hard to extract amid complex backgrounds. Conventional anchor-based models adopt fixed anchor boxes trained on limited datasets, failing to adapt to diverse real-world crack scales, which degrades generalization and accuracy while generating redundant computation. Secondly, existing networks cannot strike a balance between precision and computational cost [8]. CNNs only capture local features and lack long-range dependency modeling; transformer models with global self-attention incur heavy overhead. Window-based transformers cut computation yet weaken global feature representation, and scarce civil engineering crack datasets further hurt transformer generalization. Finally, most models are trained on handheld camera images and ignore unique imaging defects of unmanned platforms [7]. Motion blur and noise induced by device vibration and rapid movement drastically degrade detection performance when such models are deployed directly on degraded field images.

Furthermore, from an engineering deployment perspective, the three mainstream deep learning branches each carry obvious drawbacks for edge unmanned inspection. CNN classification cannot locate or quantify cracks; most object detectors lack lightweight optimization; semantic segmentation brings excessive computation and slow inference. These shortcomings fail to satisfy the low-computation and real-time constraints of dynamic unmanned monitoring, driving research on lightweight edge-oriented crack detection algorithms.

In summary, both manual inspection and existing intelligent detection techniques face prominent technical bottlenecks and cannot support large-scale intelligent structural health monitoring for modern buildings. This motivates continuous optimization of crack detection algorithms toward lightweight, high-precision unmanned inspection solutions.

### 2.2. Crack Detection Methods for Unmanned Platforms

With the rapid development of unmanned platform technologies such as unmanned aerial vehicles and unmanned vehicles, they provide an efficient and non-contact data collection solution for crack detection in building structures. Dissanayake et al. [29] proposed combining unmanned aerial vehicles with sonar sensors and visual sensors to achieve the detection and recognition of building cracks based on convolutional neural network (CNN) technology. However, at the same time, the operational characteristics of unmanned platforms also bring new challenges to detection technology.

Firstly, during the inspection process, unmanned platforms are affected by factors such as fast movement speed, machine body vibration, light changes, and complex weather. As a result, the collected crack images are prone to quality defects such as motion blur, noise interference, and uneven lighting [30]. Directly applying existing deep learning models will significantly reduce the detection accuracy.

Meanwhile, the computing resources, storage capacity, and power consumption of the embedded system of unmanned platforms are strictly constrained. Commonly used models such as GTAU-Net [27] are too complex to be directly deployed, and algorithm adaptation needs to be achieved through lightweight design. For example, the Res-RFB feature fusion module designed by Long et al. [31] for YOLOv7-tiny has improved the detection speed, but it cannot be adapted to small mobile devices due to the excessive number of parameters. The cascading group attention mechanism constructed by Zhu et al. [32] is lightweight but fails to achieve cross-node weight alignment, resulting in insufficient feature extraction stability in complex scenarios.

In addition, most existing crack datasets are constructed based on natural scene images captured by handheld cameras. The amount of building crack image data collected by unmanned platforms is limited, and the scene distribution is uneven, resulting in insufficient generalization ability of the model. The cross-domain detection accuracy is difficult to guarantee, which has become a key bottleneck restricting the implementation of crack detection technology on unmanned platforms.

### 2.3. Existing Challenges

Through a systematic review of the crack identification methods based on deep learning and the crack detection methods for unmanned platforms, the current research still faces core technical bottlenecks and challenges in the field of building crack detection on unmanned platforms:

1. How to balance model lightweighting and detection accuracy? Network pruning and parameter reduction for edge deployment inevitably degrade feature extraction capacity, especially for faint and blurry microcracks. This intrinsic trade-off inside network architectures cannot be resolved by simple structural adjustments.
2. How to solve the problem of insufficient robustness of crack features in complex scenarios? In the scenarios of building crack detection, there are complex working conditions such as variable lighting, interference from stains, crack occlusion, and irregular shapes, which lead to the interference of crack features and reduced recognition. Moreover, the interference varies greatly among different scenarios, making it difficult to adapt a single method to all scenarios and resulting in high generalization difficulty.
3. How to solve the problems of insufficient small-sample adaptation ability and weak cross-domain generalization performance of the model? The cost of collecting crack samples is high, and the characteristics of cracks made of different materials vary significantly. With small sample sizes, the model cannot fully learn the features. When applied across fields, there is a shift in feature distribution, which can easily lead to overfitting and a sudden drop in accuracy, making adaptation difficult.
4. How to resolve engineering deployment incompatibility on unmanned edge platforms? Edge hardware is constrained by limited computing power, storage capacity, and power consumption, which conflicts with the inherent high resource overhead of deep learning models. Even lightweight networks face unstable long-term inference speed and memory accumulation during continuous mobile inspection, restricting practical on-site application.

The above-mentioned technical bottlenecks have pointed out the direction for the research of this paper. Subsequent studies will address the above issues and conduct research on lightweight and highly robust crack detection algorithms for unmanned platforms, achieving a coordinated improvement in algorithm accuracy and deployment efficiency.

## 3. Methodology

### 3.1. Overview

To answer the questions above, our paper proposes a lightweight algorithm for building surface crack detection named LDD-YOLOv11, which targets unmanned mobile inspection platforms, and the overall technological framework is shown in Figure 1.

Specifically, LDD-YOLOv11 mainly consists of the original framework of YOLOv11 (Backbone → Neck → Head), the Light-Material Aware Adapter (LMA) module, the Direction-Material Constrained Focus (DMC-Focus) module, and the Dual Perception Focusing Distillation (DPFD) module.

Firstly, for the input images, the original YOLOv11 module extracts the key information of the width, the height, and the RGB three channels of the candidate crack target I as input $I\in {\mathbb{R}}_{H\times W\times 3}$ and outputs a candidate collection D:

(1) $$D={\left\{\left(b_{i},s_{i}\right)\right\}}_{i=1}^{N}$$

(2) $$b_{i}=\left(x_{i},y_{i},w_{i},h_{i}\right)$$

Here, $b_{i}$ is the bounding box is; N is the total number of crack instances detected; $x_{i},y_{i}$ are the horizontal candidates and the vertical candidates of the left-top point of the bounding box; $w_{i},h_{i}$ are the weight and the height of the bounding box, respectively.

Next, to improve the detection capability of the algorithm, we further introduce an LMA module and a DMC module into the original YOLOv11 backbone in order to reduce the influence of complex external environments and noise and also to improve the whole network’s ability to capture and recognize crack texture features and structural details.

Eventually, with respect to the problems of module performance decline and field-to-field detection accuracy deficiency in small-sample training, this study adds the DPFD module, which has the abilities of automatic recognition and target capture of high-value samples and achieves target distillation of crack detection knowledge. After integrating the high-value knowledge of distillation, the module can effectively enhance the robustness and generalization abilities of detection and also meet the ability of real-time interference edge to edge.

Then, in the following sections, we will introduce the basic principles of the LDD-YOLOv11, deconstruct its principles, and explain its operation logic.

### 3.2. LDD-YOLOv11 Algorithm

Aiming at crack detection, this paper optimizes the full pipeline of YOLOv11, covering the backbone, neck, and detection head. Figure 2 depicts the standard three-stage YOLOv11 architecture, consisting of a backbone, a neck, and a head, which sequentially completes feature extraction, feature fusion, target localization, classification, and regression. On this baseline, we embed the proposed LMA and DMC-Focus modules into the backbone and integrate the DPFD module to build the improved network, as visualized in Figure 3.

Figure 2 illustrates the standard three-stage YOLOv11 baseline consisting of a backbone, neck, and detection head, which relies on stacked C3k2 blocks for multi-scale feature extraction and fusion. Built on this architecture, our proposed LDD-YOLOv11 (Figure 3) incorporates three custom-designed components for engineering crack detection optimization.

First, as shown in Figure 3, the LMA modules are inserted at the input end and each stage of the backbone to perform illumination adaptation and texture feature enhancement. The DMC-Focus modules are embedded after the C3k modules of the backbone to strengthen directional edge features of cracks. After multi-scale feature extraction, the SPPF and upgraded C2PSA modules conduct cross-scale context aggregation and dual-channel spatial attention enhancement.

Second, the neck adopts a revised Pyramid Aggregation Spatial Pyramid Network (PASPN) structure. As an enhanced feature fusion pyramid, it hierarchically integrates high-resolution shallow crack details and high-level semantic deep features through top–down and bottom–up pathways and mitigates the feature loss of tiny cracks via Concat operations and C3k2 blocks.

Third, the decoupled anchor-free detection head separates classification and regression tasks to balance inference speed, localization precision, and lightweight network size.

Lastly, the DPFD module is introduced to transfer high-value feature knowledge from a large teacher YOLOv11s model to the lightweight student LDD-YOLOv11. The DPFD strategy is only introduced as an auxiliary loss during training and does not participate in inference calculation, so it will not increase the deployment overhead of the model. This compensates for accuracy degradation caused by network compression, improves cross-scene generalization, and enables stable real-time inference on edge unmanned inspection platforms.

#### 3.2.1. LMA: Light-Material Aware Adapter

As a core visual enhancement module of the network, the Light-Material Aware Adapter (LMA) is named for its ability to perceive both illumination conditions and surface texture features in RGB images. It should be clarified that the term “Material” here and in the subsequent Direction-Material Constrained Focus (DMC-Focus) module refers exclusively to the apparent texture and grayscale distribution characteristics of building surfaces in visible-light images rather than physical material properties or multi-modal physical sensing signals. All proposed enhancement modules operate purely on visual features extracted from RGB images.

The original YOLOv11 network framework adopts a three-phase architecture of “Backbone → Neck → Head,” and the convolutional layers within its backbone have obvious defects in the feature encoding process for crack detection. Fine cracks merely span 1–3 pixel blocks with extremely low inherent contrast. Under strong light, water reflections, dark surroundings, and cluttered backgrounds, crack textures are readily covered by noise. The root cause lies in the fact that the convolutional layers of YOLOv11’s original backbone mix and encode illumination, background, and crack texture features together, without targeted processing for illumination variations and material discrepancies. This causes crack features to appear overly dark or bright under fluctuating lighting and generates attention drift under high-frequency noise interference, which ultimately results in missed detections. To this end, we introduce the LMA as a core optimization component that is specifically designed to perceive and adapt to illumination changes and material properties within an image. By separating interference features from effective crack textures and precisely capturing fine crack information, the LMA module forms a complementary relationship with the original YOLOv11 architecture, effectively elevating the overall detection accuracy, reducing missed detections, and further improving performance.

First, drawing on Retinex theory [33], this paper decomposes the image to be detected as follows:

(3) $$I\left(x\right)=L\left(x\right)\cdot R\left(x\right)$$

where L(x) denotes the slowly varying illumination, and R(x) represents the reflectance related to texture, background, and so on.

Based on this decomposition logic, we design the LMA module to separately model illumination and texture features. The overall structure of the LMA module is visualized in Figure 4.

The input of the LMA module is feature information extracted from input images, and its output is enhanced feature representations processed through three internal core branches. This module consists of three core components—the main feature extraction path, the luminance-aware branch, and the multi-prototype modulation mechanism—which are, respectively, responsible for extracting core crack features, perceiving illumination variations in the image, and modulating features to adapt to the material characteristics of cracks. As illustrated in Figure 4, the specific details are as follows:

1. Main Feature Extraction Path. A standard Conv−BN−SiLU pipeline is employed to transform the input features:

(4) $$F_{\mathrm{base}}=\sigma \left(\mathrm{BN}\left({\mathrm{Conv}}_{3\times 3}\left(X\right)\right)\right)\in {\mathbb{R}}_{B\times C_{\mathrm{out}}\times H\times W}$$

where σ(⋅) denotes the SiLU activation function, and Conv 3×3 represents a bias-free 3 × 3 convolutional layer.

2. Luminance-aware branch [34]. This branch estimates the distribution of illumination conditions for the input feature through global context modeling:

(5) $$w=\mathrm{Softmax}\left({\mathrm{Conv}}_{1\times 1}\left(\mathrm{GAP}\left(X\right)\right)\right)\in {\mathbb{R}}_{B\times K}$$

where GAP(⋅) denotes global adaptive average pooling, and K=3 indicates the number of prototypes, corresponding to three typical conditions: low illumination, normal illumination, and high illumination; the softmax function achieves weight normalization.

3. Multi-Prototype Residual Modulation. The module maintains a set of learnable prototype embeddings $P\in {\mathbb{R}}_{K\times C_{\mathrm{out}}}$, which are fused using illumination weights:

(6) $$s=w\cdot P\in {\mathbb{R}}_{B\times C_{\mathrm{out}}}$$

The final output is generated through channel-wise residual multiplicative modulation:

(7) $${\hat{F}}_{b,c,h,w}=F_{b,c,h,w}^{\mathrm{base}}\left(1+s_{b,c}\right)$$

where ${\hat{F}}_{b,c,h,w}$ denotes the enhanced output feature tensor, $F_{b,c,h,w}^{\mathrm{base}}$ is the base input feature tensor, and *s_b,c_* stands for the learned modulation scalar of the c-th channel. This expression implements channel-wise multiplicative modulation in standard element-wise notation: each per-channel scalar is applied to every spatial position (height and width) of the corresponding feature channel via Hadamard product. The residual form (1 + *s*) ensures the identity mapping property at the initialization stage, which is conducive to stable training convergence.

Aiming at crack detection across diverse illumination scenarios, two problems readily manifest: Crack features in nighttime or dim tunnel scenes are excessively weak, and crack features near strong light or reflective materials are prone to being overwhelmed. Accordingly, the LMA module leverages dark, normal, and bright illumination prototypes to explicitly encode typical lighting patterns. It extracts the illumination feature distribution of input images to calculate global and regional illumination levels and then adaptively characterizes light variations via the three illumination prototypes [35].

For each spatial position (i,j), extract the local illumination descriptor $z_{i,j}\in {\mathbb{R}}^{D}$ using a lightweight context encoder ϕ(·):

(8) $$z_{i,j}=\phi ({\mathrm{Pool}}_{\Omega (i,j)}(X))\;$$

Ω(i,j) denotes a local window centered at (i,j), where the notation ϕ(·) represents a combination of a 1×1 convolution and a small MLP.

It then identifies the illumination prototype weights for different regions. The membership weight of position (i,j) for the k-th illumination prototype is calculated using softmax normalization with a temperature coefficient:

(9) $$w_{k}(i,j)=\frac{\exp\left(\tau^{-1}\langle z_{i,j},p_{k}\rangle \right)}{\sum_{k^{\prime }=1}^{K}\exp\left(\tau^{-1}\langle z_{i,j},p_{k^{\prime }}\rangle \right)}\;$$

$\langle \cdot ,\cdot \rangle$ denotes the cosine similarity, with τ being the temperature coefficient that regulates distribution sharpness. According to the properties of softmax, it follows that $\sum_{k=1}^{K}w_{k}(i,j)=1$.

If the weight of the dark prototype is relatively large, the crack-related channel features in that region are amplified to brighten the crack features in dark areas; if the weight of the normal prototype is large, virtually no adjustment is made; if the weight of the bright prototype is relatively large, the illumination channel features in that region are reduced while the crack channel features are enhanced, thereby achieving strong-light and reflection suppression together with crack feature enhancement.

The weight adjustment is implemented via the residual adaptive modulation function $\hat{F}=F⊙(1+s)$, where s refers to the channel modulation vector, which is adaptively generated for the illumination condition of each region through a softmax assignment mechanism:

(10) $$s=\sum_{k=1}^{K}w_{k}\cdot p_{k},\;\sum_{k=1}^{K}w_{k}=1,\;w_{k}\geq 0$$

This vector has excellent interpolation properties, facilitating smooth transitions between two illumination levels. The weight adjustment allows better normalization of global illumination while highlighting invariant feature channels like crack textures and directions, ensuring that the input information’s crack features are unbiased by illumination variation.

Let $C_{\mathrm{in}},C_{\mathrm{out}}$ be the numbers of input and output channels, respectively, let k be the convolution kernel size, let H and W denote the height and width of the feature map, and let B represent the batch size. The LMA module comprises a standard Conv−BN component and a newly added lightweight branch, and its specific parameter count is given by the following formula:

(11) $$\left\{\begin{array}{l} \Theta_{\mathrm{Conv}-\mathrm{BN}}=C_{\mathrm{in}}C_{\mathrm{out}}k^{2}+2C_{\mathrm{out}}\\ \Theta_{\mathrm{add}}=C_{\mathrm{in}}C_{\mathrm{out}}+C_{\mathrm{out}}K \end{array}\right.$$

In Equation (9), the parameter count of the standard Conv−BN component is denoted as $\Theta_{\mathrm{Conv}-\mathrm{BN}}$; the parameter count of the newly added lightweight branch is denoted as $\Theta_{\mathrm{add}}$.

Hence, the total parameter count of the LMA module is

(12) $$\Theta_{\mathrm{LMA}}=C_{\mathrm{in}}C_{\mathrm{out}}k^{2}+2C_{\mathrm{out}}+C_{\mathrm{in}}C_{\mathrm{out}}+C_{\mathrm{out}}K$$

A complexity analysis of the LMA module is conducted, and the specific calculations are given by the following formulas:

(13) $$\left\{\begin{array}{l} {\mathrm{FLOPs}}_{\mathrm{main}}=O\left(C_{\mathrm{in}}C_{\mathrm{out}}k^{2}HW\right)\\ {\mathrm{FLOPs}}_{\mathrm{om}}=O\left(C_{\mathrm{in}}K+BKC_{\mathrm{out}}\right)\\ {\mathrm{FLOPs}}_{l}=O\left(C_{\mathrm{in}}HW\right)\\ {\mathrm{FLOPs}}_{w}=O\left(BC_{\mathrm{out}}HW\right) \end{array}\right.$$

where ${\mathrm{FLOPs}}_{\mathrm{main}}$ refers to the FLOPs of the main convolution; ${\mathrm{FLOPs}}_{1}$ corresponds to the FLOPs of prototype mapping and prototype fusion; ${\mathrm{FLOPs}}_{\mathrm{om}}$ denotes the FLOPs of the illumination branch; and ${\mathrm{FLOPs}}_{w}$ represents the FLOPs multiplying the attention weights with the feature map.

Therefore, the total FLOPs of the LMA module are

(14) $${\mathrm{FLOPs}}_{\mathrm{LMA}}=C_{\mathrm{in}}C_{\mathrm{out}}k^{2}HW+C_{\mathrm{in}}HW+C_{\mathrm{in}}K+BKC_{\mathrm{out}}+BC_{\mathrm{out}}HW$$

#### 3.2.2. DMC-Focus: Direction-Material Constrained Focus

To further strengthen the feature extraction capability for fine cracks and to address the difficulty of distinguishing crack features under complex interference, based on the optimization provided by the LMA module and in combination with the inherent core geometric features of cracks, we design the DMC-Focus module. Through a dual-branch mechanism of direction constraint and material constraint, it achieves precise focusing on crack features and improves crack detection performance.

The DMC-Focus module is composed of three cascaded sub-modules: the direction field perception branch, the reflection feature enhancement module, and the focus weight generator, which separately capture crack orientation, boost crack reflectance contrast, and generate attention weights to highlight valid crack features [36]. It realizes dual constraint mechanisms for noise suppression and faint crack enhancement. The direction branch uses directional convolution kernels to estimate regional crack directions and confidence, while the reflection branch reuses LMA’s illumination-reflectance results to eliminate highlight interference. Their fused outputs form a focus attention map to reinforce crack textures. Cooperating with the LMA module, it remarkably elevates the detection precision of tiny cracks. The complete structure is demonstrated in Figure 5, and its core branches are detailed as follows:

Firstly, regarding the direction field perception branch. Cracks exhibit elongated, continuously oriented geometric characteristics. This branch predicts pixel-wise direction fields over input feature maps [37] via the following formula:

(15) $$D=\tanh\left({\mathrm{Conv}}_{3\times 3}\left(\sigma \left(\mathrm{BN}\left({\mathrm{Conv}}_{3\times 3}\left(X\right)\right)\right)\right)\right)\in {\mathbb{R}}_{B\times 2\times H\times W}$$

where the channel dimension is 2, corresponding to the horizontal component $d_{x}$ and the vertical component $d_{y}$; the tanh function ensures that the direction component values range within [−1,+1]; the number of channels in the intermediate hidden layer is set to $\max\left(\frac{C}{4},8\right)$, striking a balance between expressiveness and efficiency.

Secondly, for the reflection feature enhancement module. Since each feature channel corresponds to distinct boundary patterns, depthwise separable convolutions are used to process high-frequency signals per channel and suppress false boundaries caused by background highlights:

(16) $$E=\mathrm{BN}\left(\mathrm{PW}\left(\sigma \left(\mathrm{BN}\left({\mathrm{DW}}_{3\times 3}\left(F_{\mathrm{ref}}\right)\right)\right)\right)\right)\in {\mathbb{R}}_{B\times C\times H\times W}$$

where ${\mathrm{DW}}_{3\times 3}$ denotes a 3 × 3 depthwise convolution, and PW represents a 1 × 1 pointwise convolution [38].

Thirdly, the focus weight generator. The extracted direction field and reflection boundary features are concatenated along the channel dimension and then fed into a lightweight bottleneck structure to output a joint spatial-channel attention map:

(17) $$A=\mathrm{Sigmoid}\left({\mathrm{Conv}}_{1\times 1}\left(\sigma \left(\mathrm{BN}\left({\mathrm{Conv}}_{1\times 1}\left(\left[E,D\right]\right)\right)\right)\right)\right)\in {\left[0,1\right]}_{B\times C\times H\times W}$$

where [E;D] represents channel concatenation, and the input possesses C+2 channels. The final output is obtained via residual weighting:

(18) $$\hat{X}=X+X⊙A$$

The residual connection ensures smooth gradients, and the Sigmoid function of A constrains the gain range at each position to [1,2], meaning that features are only enhanced and never suppressed.

DMC-Focus employs a dual mechanism of direction constraint and reflection constraint to jointly suppress specular pseudo-boundaries:

In terms of direction constraint, cracks, as products of physical field actions, obey mechanical and physical laws and exhibit directional consistency. In contrast, for junctions of materials and construction interfaces, such as asphalt seam lines on roads or brick gaps, their direction fields demonstrate abrupt changes and are relatively orderly and regular. The direction branch imposes control through a loss function:

(19) $$L_{\mathrm{dir}}=\lambda_{\mathrm{dir}}\cdot \frac{1}{2HW}\sum_{c\in \{x,y\}}\left(\left|\nabla_{x}D_{c}\right|+\left|\nabla_{y}D_{c}\right|\right)$$

where $\lambda_{\mathrm{dir}}$ is a hyperparameter controlling the strength of this loss; H and W are the height and width of the image; $\nabla_{x},\nabla_{y}$ are the horizontal and vertical gradients, respectively, approximated via the Sobel operator; and $D_{c}$ is the component of the direction field D in the x or y direction. It determines whether the direction field in a region is smooth and continuous, discarding parts with large gradients while preserving those with small gradients, thereby controlling the smoothness of the direction field to differentiate the confidence of cracks. Regions with high confidence receive increased attention, while those with low confidence receive reduced attention, thereby diminishing material and noise interference and improving crack recognition accuracy and robustness.

In terms of reflection constraints, real cracks possess a stably varying gradient from the boundary inward, whereas specular reflection boundaries, unlike real crack boundaries, often exhibit abrupt gradient changes. The DMC-Focus module adjusts attention weights according to the degree of gradient abruptness [39,40].

The above direction constraint and reflection constraint complement and coordinate with each other in the feature space. The direction branch primarily handles interference at the geometric level, while the reflection branch mainly addresses interference at the optical level, and together, they achieve further precise recognition of cracks.

To quantitatively evaluate the computational overhead of the proposed DMC-Focus module, this paper analyzes it using standard parameter estimation methods employed in convolutional neural networks. Specifically, a 2D convolutional layer with a kernel size of k×k, $C_{in}$ input channels, and $C_{out}$ output channels contains $C_{in}\cdot C_{out}\cdot k^{2}+C_{out}$ parameters (including biases), while a batch normalization layer comprises $2C_{out}$ learnable parameters. Based on the DMC-Focus architecture described in the previous section (utilizing 3×3 convolutions with channel compression ratios of 1/4 or 1/2), the parameter quantities for its three branches can be derived as follows.

The direction field perception branch first compresses input features from C channels to C/4 channels before outputting a dual-channel direction field, with a total parameter count of

(20) $$\Theta \left(C\cdot \frac{C}{4}\cdot 9+2\cdot \frac{C}{4}+\frac{C}{4}\cdot 2\cdot 9+2\right)$$

The focus weight generator consists of a 3 × 3 deep convolution and a lightweight channel attention module cascaded together, with a total number of parameters of

(21) $$\Theta \left(C\cdot 9+2C+C^{2}+2C\right)$$

The reflection feature enhancement module combines the two-channel reflection rate map with input features and feeds it into a bottleneck structure with a compression ratio of 1/2, for which its number of parameters is

(22) $$\Theta =((C+2)\cdot \frac{C}{2}+2\cdot \frac{C}{2}+\frac{C}{2}\cdot C+C)$$

Thus, the total parameter count for the DMC-Focus module is

(23) $$\Theta =\frac{17}{4}C^{2}+21C+2$$

The term resembling $C_{in}\cdot C_{out}\cdot 3^{2}$ corresponds to a 3×3 convolution, where coefficient 2 represents either the affine parameter (γ,β) of the BN layer or the bias vector. It should be noted that Equations (18)–(20) are not universal formulas but are directly derived from the general convolution parameter calculation rules combined with the specific architecture of DMC-Focus designed in previous sections; if the convolution kernel size or channel compression ratio changes, their coefficients will accordingly vary.

An analysis of the computational complexity of the DMC-Focus module is conducted. DMC-Focus combines depthwise convolution and pointwise convolution to construct depthwise separable convolutions, supplemented by several lightweight linear operations, feature transformation operations, and loss calculations. Its FLOPs are computed according to the following formulas:

(24) $$\left\{\begin{array}{l} {\mathrm{FLOPS}}_{\mathrm{DSC}}=2C\cdot \frac{C}{4}\cdot 9\cdot HW\;\\ {\mathrm{FLOPS}}_{\mathrm{LW}}=C\cdot 9\cdot HW\;\\ {\mathrm{FLOPS}}_{\mathrm{FT}}=C_{2}\cdot HW+(C+2)\cdot C\cdot HW\; \end{array}\right.$$

In the given formula, ${\mathrm{FLOPs}}_{\mathrm{DSC}}$ denotes the primary computation of the depthwise separable convolution; ${\mathrm{FLOPs}}_{\mathrm{Lw}}$ and ${\mathrm{FLOPs}}_{\mathrm{FT}}$ correspond to feature transformation operations and the loss.

Therefore, the total computation for the DMC-Focus module is

(25) $${\mathrm{FLOPs}}_{\mathrm{DMC}}\approx 2C\cdot \frac{C}{4}\cdot 9\cdot HW+C\cdot 9\cdot HW+C_{2}\cdot HW+(C+2)\cdot C\cdot HW$$

Taking input C=128, H=W=80 as an example, ${\mathrm{FLOPs}}_{\mathrm{DMC}}\approx 5.9\times 10^{8}\;\mathrm{FLOPs}$, comparable to a single-layer C3k2 module in the backbone network with $5.6\times 10^{8}\;\mathrm{FLOPs}$, which is considered an acceptable computational overhead.

### 3.3. DPFD: Dual Perception Focusing Distillation

After feature extraction enhancement via LMA and DMC-Focus, crack detection tasks face two practical challenges: Limited labeled data restricts the model’s ability to learn diverse crack features and weakens cross-domain generalization. To tackle this issue, we propose the DPFD module with dual uncertainty-similarity perception for targeted knowledge distillation.

Conventional distillation assigns equal weight to all samples, ignoring sample disparities. For crack detection, hard low-confidence samples with tiny, blurry cracks and similar crack-feature samples carry vital training information. DPFD automatically screens these two high-value sample groups and conducts focused knowledge distillation. Unlike indiscriminate full distillation, it boosts knowledge transfer efficiency, elevating detection accuracy for complex scenes and cross-domain cracks. Combined with LMA and DMC-Focus, the module jointly improves the baseline YOLOv11’s precision and generalization.

In this study, its specific framework is illustrated in Figure 6. This mechanism takes the YOLOv11s large model pre-trained on the source domain and fully freezes it as the teacher network and the LDD-YOLOv11 lightweight model fine-tuned on the target domain as the student network. Specifically, the teacher model is pre-trained exclusively on the public CCIC crack dataset to learn general crack feature priors, while the student LDD-YOLOv11 is trained solely on our self-constructed Nanjing building crack dataset. The CCIC dataset is only used for teacher model pre-training and does not participate in the training process of the student model. This strict data division ensures that the observed distillation improvement comes purely from cross-domain knowledge transfer rather than data leakage between training sets.

Its inputs include multi-scale feature maps $F_{t}$, soft label distributions $L_{t}$, and a pre-constructed source-domain prototype library M output by the teacher side from the source-domain pre-trained model, as well as corresponding feature maps $F_{s}$ and prediction distributions $L_{s}$ from the trainable lightweight student model. On this basis, a three-tier distillation link is constructed, achieving efficient transfer of the teacher network’s strong feature representation capability to the student network through feature-level distillation, response-level distillation, and prototype contrastive distillation.

Specifically, feature-level distillation employs L2 normalization and MSE (Mean Squared Error) loss $L_{feat}$ to align the multi-scale feature distributions of the teacher and student networks; response-level distillation introduces a temperature coefficient T=4.0 to soften the teacher’s soft labels and the student’s predicted distributions, and then, it transfers class probability knowledge through KL divergence loss $L_{resp}$ to reduce the impact of label noise; prototype contrastive distillation is constructed based on InfoNCE loss $L_{con}$ to impose similarity constraints between student features and the source-domain prototype library, thereby enhancing cross-domain generalization capability.

Simultaneously, to balance distillation and detection tasks, the mechanism introduces a dynamic weight scheduling strategy, dividing the training process into three stages: The initial stage uses only the detection loss to build the student network’s basic recognition capability; the middle stage linearly increases the distillation weight to gradually introduce teacher supervision information; the later stage stabilizes the weight to reinforce the guidance of teacher knowledge to the student model. Ultimately, the overall loss function $L_{\mathrm{total}}=\alpha \cdot L_{\mathrm{feat}}+\beta \cdot L_{\mathrm{resp}}+\gamma \cdot L_{\mathrm{con}}$ enables end-to-end optimization of the entire network. The hyperparameters α, β, γ are set based on the rationale that feature-level distillation is the core step of knowledge transfer, directly determining the student model’s ability to represent fine crack details; hence, it is assigned the highest weight α; response-level distillation serves as an auxiliary calibration for class probabilities and is assigned a weight β to avoid conflict with the detection task. Prototype contrastive distillation, as a supplementary constraint, mainly aims to improve cross-domain generalization performance and is assigned a weight γ to prevent excessive guidance that may cause adaptation bias in the target domain. The rationality of the default weight values and the robustness of model performance to hyperparameter adjustments are further verified through single-factor controlled variable experiments in Section 4.3.4.

During the inference stage, the teacher model and all distillation modules are completely removed, introducing no additional computational latency, thereby significantly compressing the model parameter count while ensuring detection accuracy and meeting the real-time deployment requirements of edge devices.

DPFD defines distillation targets at three levels of granularity:

1. Feature-Level Distillation: The intermediate-layer features of the student and teacher networks are aligned via MSE [41] to obtain the following loss:

(26) $$L_{\mathrm{feat}}=\mathrm{MSE}(\hat{\phi }(F_{S}),\hat{\phi }(F_{T}))$$

where $\hat{\phi }\left(\cdot \right)$ denotes the L2 normalization operation. When the teacher and student channel numbers differ, alignment is performed via a 1 × 1 learnable projection layer $W_{\mathrm{align}}\in {\mathbb{R}}_{C_{T}\times C_{S}}$, and spatial mismatches are handled by adaptively averaging pools on student features.

2. Response-Level Distillation. For the logits of the teacher and student models, the output distributions $Z_{T},Z_{S}$ use temperature-scaled KL divergence:

(27) $$L_{\mathrm{resp}}=T_{2}\cdot D_{\mathrm{KL}}\left(\mathrm{softmax}\left(\frac{Z_{T}}{T}\right)∥\mathrm{softmax}\left(\frac{Z_{S}}{T}\right)\right)$$

where temperature T=4.0 compensates for the gradient attenuation induced by temperature scaling.

3. Prototype Contrastive Distillation: Based on structured knowledge transfer from the prototype library $P\in {\mathbb{R}}_{K\times D}$,

(28) $$L_{\mathrm{contrast}}=D_{\mathrm{KL}}\left(\mathrm{softmax}\left(\frac{F_{T}P^{T}}{T}\right)∥\mathrm{softmax}\left(\frac{F_{S}P^{T}}{T}\right)\right)$$

The total distillation loss is the weighted sum of the three terms:

(29) $$L_{\mathrm{distill}}=\alpha L_{\mathrm{feat}}+\beta L_{\mathrm{resp}}+\gamma L_{\mathrm{contrast}}$$

with parameters being α=1.0,β=0.5,γ=0.3. (The appropriateness of the parameter settings will be demonstrated in Section 4.3.4.)

To prevent the distillation signals from interfering with the convergence of the student model during the early training phase, a three-stage weight scheduling strategy is adopted:

(30) $$(w_{\mathrm{std}}(t),w_{\mathrm{dist}}(t))=\left\{\begin{array}{ll} \left(1.0,0.0\right) & t<t_{\mathrm{warm}}\\ \left(1.0-ap,bp\right) & t_{\mathrm{warm}}\leq t<t_{\mathrm{warm}}+t_{\mathrm{stable}}\\ \left(1.0-a,b\right) & t\geq t_{\mathrm{warm}}+t_{\mathrm{stable}} \end{array}\right.$$

where t denotes the current training time step, and $t_{warm}$ and $t_{stable}$ are the warm-up and stabilization step counts, respectively, with $\rho =\frac{t-t_{\mathrm{warm}}}{t_{\mathrm{stable}}}$ being the linear progress factor during the stabilization stage. a,b∈[0,1] are scheduling coefficients used to control the final weight ratio between student loss and distillation loss during the later stages of training; both are empirically determined; see Section 4.1.3 for specific values. This design allows the student model to first establish a preliminary weight foundation by relying solely on its own loss during the warm-up period, thereby avoiding instability caused by source-domain information interference in the early stage; as training progresses, the distillation knowledge from the teacher model is gradually introduced, assisting the student model in absorbing the experience embedded in the prototype library; upon reaching the stable period, the student model continues to learn under the steady supervision of the teacher model, reinforcing its feature consistency with the teacher.

To achieve efficient knowledge distillation and model optimization, we inherit the Ultralytics BaseTrainer and perform a dynamically weighted total loss computation at each training step:

(31) $$L_{\mathrm{total}}(t)=w_{\mathrm{std}}(t)\cdot L_{\det}+w_{\mathrm{dist}}(t)\cdot L_{\mathrm{distill}}$$

where $L_{\det}$ is the base task loss for crack detection, and $L_{\mathrm{distill}}$ is the multi-granularity total distillation loss defined earlier. $W_{\mathrm{std}}(t)$ and $W_{\mathrm{dist}}(t)$ are the dynamic weights for the corresponding losses at training step t. Throughout the entire training process, the teacher model is kept frozen and operates only in inference mode to provide knowledge support.

Such soft labels provide richer supervisory knowledge and confidence cues for model distillation, enabling the student model to capture fine-grained feature characteristics similar to real crack patterns [42]. For example, a teacher’s soft label with probabilities of multiple categories implicitly contains refined decision information. This “dark knowledge” effectively improves the knowledge density of individual training samples, equivalently expanding the valid data scale and enhancing the feature learning ability of lightweight student models.

To guarantee effective knowledge transfer, the DPFD framework adopts dual distillation loss constraints. The feature distillation loss eliminates biased feature learning in the target domain, while the prototype contrastive loss enables the student model to efficiently inherit high-value teacher knowledge and maintain consistent feature category distribution. Cooperating with its three-stage progressive distillation design, DPFD allows the student model to steadily absorb teacher domain knowledge after basic weight convergence. This progressive injection avoids excessive interference from source-domain redundant features and ensures effective transfer of target-domain crack knowledge. Benefiting from the above mechanisms, the lightweight student model achieves superior detection robustness and cross-scene generalization performance.

### 3.4. Module Synergy Mechanism

Having described the individual structures and functions of LMA, DMC-Focus, and DPFD, this section further analyzes the synergistic mechanism among the three modules and explains the source of the nonlinear performance gain that exceeds the simple sum of individual contributions.

Instead of working as parallel independent components, the three modules form a serial progressive complementary pipeline from input preprocessing and backbone feature extraction to training optimization. The synergistic relationship is illustrated in Figure 7 and elaborated as follows.

At the front end of the pipeline, the LMA module acts as a global visual preprocessing unit. It suppresses uneven illumination, highlight reflection, and background luminance noise in the input RGB image and improves the signal-to-noise ratio (SNR) of crack texture features before they enter the backbone network. This step provides high-quality clean input for subsequent feature extraction and is a prerequisite for the effective operation of downstream fine-grained feature enhancement modules.

In the middle of the backbone, the DMC-Focus module performs feature-level enhancement based on visual edge patterns. It enhances the directional edge features of cracks and filters pseudo-crack background interference such as brick joints, stains, and texture noise. Notably, the performance of DMC-Focus is highly dependent on the denoising preprocessing of the front-end LMA. Without LMA to eliminate global illumination clutter, complex background noise will severely interfere with the directional feature extraction of DMC-Focus and cause a large number of false positive responses on non-crack edges. This dependency is the core reason why the combination gain of LMA and DMC-Focus exceeds the sum of their individual gains.

At the training stage, the Dual Perception Focused Distillation (DPFD) strategy further amplifies the feature advantages of the first two modules from the optimization level. It injects the crack prior knowledge learned by the large teacher model into the lightweight student model and guides the student network to pay more attention to the weak texture features enhanced by LMA and DMC-Focus. In this way, the feature enhancement effect of the front two modules is fully exploited during training, and the generalization ability of the lightweight model on cross-scene crack detection is further improved.

In general, LMA solves the input visual quality problem, DMC-Focus solves the feature discrimination problem, and DPFD solves the training optimization efficiency problem. The three modules cooperate layer by layer, and the performance improvement of each module will be transmitted and amplified through subsequent links, thus forming a nonlinear synergistic gain. Quantitative verification of this synergistic effect is provided in the ablation experiments in Section 4.3.1.

## 4. Experimental Studies

### 4.1. Experimental Setup

#### 4.1.1. Dataset Introduction

The public dataset used in knowledge distillation, the Concrete Crack Images for Classification (CCIC), was published by Çağlar Fırat Özgenel [43]. This dataset contains 40,000 images gathered from various building structures across the METU campus in Turkey. It provides robust data support for crack detection model distillation tasks within this study. We selected 10,000 crack-containing images as experimental samples. Original images have a resolution of 1920 × 1080, which we resized to 640 × 640 to accommodate model input requirements, as shown in Figure 8.

To evaluate the performance of the proposed LDD-YOLOv11, we constructed a real-world building crack dataset containing 5000 images from 50 independent buildings in Nanjing, China. All images are captured by consumer-grade digital cameras and resized to 640 × 640 pixels. Crack instances are manually annotated with bounding boxes by professional inspectors.

To avoid data leakage caused by random image-level splitting, we adopt building-level dataset partitioning instead of the conventional random split strategy. Specifically, images from the same building or wall surface are exclusively assigned to either the training set or the test set, with no overlapping scenes between the two subsets. The training set contains 4012 images from 36 buildings, and the test set contains 988 images from 14 independent buildings.

Table 1 summarizes the detailed statistics of the dataset in terms of crack width levels, illumination conditions, and background interferences. The dataset covers diverse real-world scenarios, including ultra-fine cracks, strong illumination reflections, and various background clutter, which can comprehensively validate the robustness of the detection model, as shown in Figure 9.

It should be clarified that the CCIC dataset is only used for teacher model pre-training in the DPFD paradigm; all training and evaluation of the final LDD-YOLOv11 student model are performed exclusively on the self-built building crack dataset.

#### 4.1.2. Experimental Configuration

All experiments in this study are implemented based on the PyTorch deep learning framework. The underlying system runs on the Ubuntu 22.04 operating system with the Python 3.10 interpreter. Model training is performed on NVIDIA GeForce RTX 4090 graphics cards (GPUs) with a video memory capacity of 24 GB, accelerated by the CUDA 12.1 parallel computing architecture. The input images of all models are uniformly resized to 640 × 640 pixels.

As shown in Table 2, the Stochastic Gradient Descent (SGD) optimizer is adopted for training, with a cosine annealing learning rate scheduler. A learning rate warm-up strategy is applied in the first five epochs to stabilize the training process. To improve the generalization ability of the model, standard data augmentation operations including Mosaic augmentation, MixUp augmentation, random horizontal flip, and photometric distortion (brightness, contrast, and blur) are introduced during training.

For the DPFD strategy, the hyperparameters are set following the definition in Equation (30). Specifically, the feature distillation weight α, response distillation weight β, and prototype contrast weight γ are set to 1.0, 0.5, and 0.3, respectively, yielding a final weight ratio of 1:0.8 between the base detection loss and the total distillation loss. In terms of phase durations, the preheating warm-up phase lasts five epochs, consistent with the learning rate schedule above; the linear ramp-up phase spans 95 epochs; the stabilization phase covers the remaining 200 epochs within the total 300 training epochs. All these values are determined through preliminary tuning experiments on the validation split of the training dataset, and their sensitivity and robustness are further verified through single-factor controlled experiments in Section 4.3.4.

#### 4.1.3. Evaluation Metric Consistency

To ensure fair comparison between object detection models and semantic segmentation models under the same evaluation standard, this paper uniformly adopts mAP@0.5 as the core comparison metric. The calculation rules for different types of models are described as follows.

For object detection models, including all YOLO series baselines and the proposed LDD-YOLOv11 (which uses a decoupled anchor-free detection head), mAP@0.5 is calculated directly following the standard detection evaluation protocol originally defined by the PASCAL VOC and Microsoft COCO benchmarks [44,45]. The predicted bounding boxes are matched with ground-truth bounding boxes under the Intersection over Union (IoU) threshold of 0.5, and the average precision across all crack instances is computed.

For semantic segmentation models such as U-Net, DeepCrack, and CrackFormer-II, we convert their pixel-level segmentation outputs into bounding box results before mAP calculation. Specifically, we first extract connected crack regions from the binary segmentation mask and then generate the minimum bounding rectangle (MBR) for each connected crack region through contour extraction. The generated bounding boxes are then evaluated using exactly the same mAP@0.5 calculation pipeline and IoU threshold as the detection models.

This unified box-level evaluation protocol is adopted for two reasons. First, it enables direct and fair cross-paradigm comparison between detection and segmentation methods. Second, in practical engineering inspection scenarios, the final output of crack detection is usually the regional location of defects, and box-level indicators are more consistent with the actual demand of structural health monitoring. In addition, we also report box-level IoU and F1 score for the segmentation models as auxiliary evaluation after the bounding-box conversion.

### 4.2. Evaluation Metrics

#### 4.2.1. Performance Evaluation Indicators

To comprehensively and objectively evaluate LDD-YOLOv11’s performance, the following metrics were chosen: precision (P), recall (R), F1 score (F1), and mean average precision (mAP). The formulas are as follows:

1. Precision (P):

(32) $$P=\frac{TP}{TP+FP}$$

This is the proportion of true positive samples among those predicted as positive.

2. Recall (R):

(33) $$R=\frac{TP}{TP+FN}$$

This is the proportion of true positive samples correctly predicted as positive.

3. F1 Score (F1):

(34) $$F1=2\times \frac{P\times R}{P+R}$$

These are the harmonious means reflecting the balance between precision and recall.

4. mAP:

(35) $$mAP=\frac{\sum_{i=1}^{N}\int P(R)dR}{N}$$

This is the core indicator reflecting the model’s overall correct recognition capabilities across all detection categories, where TP and FP are counts of true and false positives predicted, FN is the count of falsely predicted negatives, and N denotes the total detection task categories, which is 1 for this study.

#### 4.2.2. Results and Visual Analysis

For an intuitive demonstration of this study’s improvements, visual comparisons between pre- and post-improvement models were first conducted. Figure 10 contrasts the detection performance of the LDD-YOLOv11 algorithm (right) with the YOLOv11n algorithm (center).

Specifically, in the crack detection scenario shown in Figure 10a, the original YOLOv11n model missed some small-sized crack targets with high similarity to the background, resulting in poor confidence in the recognition effect. However, the improved model can detect all of them with higher confidence. In the long target detection scenario shown in Figure 10b, when facing images of longer length, the improved model demonstrates stronger recognition ability, and the overall detection situation is significantly better. It can be seen from the multi-object detection results in Figure 10c that the improved model can more clearly distinguish and locate the targets.

#### 4.2.3. Analysis of the Loss Function in the DPFD Module

To further interpret the training mechanism of the proposed DPFD module, we analyze its loss function, for which the detailed calculation formulas are provided as follows.

(36) $$L(t)=w_{\mathrm{std}}(t)\cdot {\hat{L}}_{\det}(y_{S},y_{\mathrm{gt}})+w_{\mathrm{dist}}(t)\cdot \left[\alpha L_{\mathrm{feat}}+\beta L_{\mathrm{resp}}+\gamma L_{\mathrm{contrast}}\right]$$

Here,

(37) $$\left\{\begin{array}{l} {\hat{L}}_{\det}(y_{S},y_{\mathrm{gt}})\\ L_{\mathrm{feat}}={\left\|\hat{\phi }(F_{S})-\hat{\phi }(F_{T})\right\|}_{2}^{2}\\ L_{\mathrm{resp}}=T_{2}D_{\mathrm{KL}}(\sigma_{T}∥\sigma_{S})\\ L_{\mathrm{contrast}}=D_{\mathrm{KL}}(S_{T}^{\mathrm{proto}}∥S_{S}^{\mathrm{proto}}) \end{array}\right.$$

where

$L_{\det}\left({\hat{y}}_{S},y_{\mathrm{gt}}\right)$ denotes the YOLO standard detection loss used for constraining the model during the early training stage.

$L_{\mathrm{feat}}={\left\|\hat{\phi }\left(F_{S}\right)-\hat{\phi }\left(F_{T}\right)\right\|}_{2}^{2}$ is the feature distillation loss, which helps the student model effectively acquire the feature knowledge of the teacher model for learning.

$L_{\mathrm{resp}}=T_{2}D_{\mathrm{KL}}\left(\sigma_{T}|\sigma_{S}\right)$ is the response distillation loss, which increases the probability of suboptimal features through the temperature T and improves model generalization.

$L_{\mathrm{contrast}}=D_{\mathrm{KL}}\left(S_{T}^{\mathrm{proto}}|S_{S}^{\mathrm{proto}}\right)$ is the prototype contrastive loss, which is used to ensure that the structural and discriminative conditions between similar categories of the teacher and student models remain consistent during the stabilized later period, thus consolidating the distillation effect.

### 4.3. Experimental Results Analysis

#### 4.3.1. Ablation Studies

Ablation experiments are conducted to quantitatively verify the individual contribution of each proposed module and the synergistic effect among them. All experiments are built upon the YOLOv11n baseline, and modules are added incrementally to compare performance changes. The core evaluation metric is mAP@0.5, and the results are presented in Figure 11.

It should be noted that all ablation configurations are trained from scratch without pre-trained weights using completely identical training hyperparameters, data augmentation, and epoch schedules. This controlled single-variable setting is designed to purely evaluate the intrinsic contribution of each module, free from the interference of general pre-trained visual features. Under this setting, the baseline YOLOv11n achieves an mAP@0.5 of 82.2%, which is used for intra-group relative comparisons of module gains.

In contrast, the comparative experiments in Section 4.3.2 adopt the standard fine-tuning protocol with official pre-trained weights, full training schedules, and complete augmentation pipelines, which follows the common evaluation convention in the field to ensure fair comparison with state-of-the-art methods. Therefore, the YOLOv11n baseline under full training reaches 88.3% mAP@0.5, which serves as the reference for cross-method performance comparison.

As shown in the results, each individual module brings consistent performance improvement compared with the baseline model (mAP@0.5 = 82.2%).

1. Adding only the LMA module raises mAP@0.5 to 84.5%, with an absolute improvement of 2.3 percentage points. This verifies that the luminance-aware and texture adaptation mechanism effectively enhances the expression of weak crack features and improves the feature capture capability of the backbone network.
2. Adding only the DMC-Focus module achieves 85.1% mAP@0.5, corresponding to a 2.9 percentage point improvement. The result indicates that the directional constrained focus mechanism plays a greater role in suppressing pseudo-crack background noise and enhancing the discriminability of crack edge features.
3. Introducing the DPFD strategy alone obtains 84.8% mAP@0.5, with a 2.6 percentage point gain. This confirms that knowledge transfer from the teacher model can effectively improve the generalization ability of the lightweight student model without modifying the inference structure.

To further explore the cooperative relationship between modules, we conduct pairwise combination experiments. The combination of LMA and DMC-Focus achieves the best dual-module performance with 85.9% mAP@0.5. The two modules form a serial progressive enhancement pipeline: LMA first suppresses global illumination noise and improves input feature quality, which creates favorable conditions for DMC-Focus to extract directional crack features. This complementary effect enables the dual-module configuration to outperform any single module. The LMA + DPFD and DMC + DPFD combinations achieve 85.6% and 85.8% mAP@0.5, respectively. Both configurations outperform single-module settings, demonstrating that the DPFD strategy can further amplify the feature advantages brought by LMA and DMC-Focus at the training optimization level.

When all three modules are integrated, the full LDD-YOLOv11 model reaches the optimal mAP@0.5 of 90.5%, with a total improvement of 8.3 percentage points over the baseline. It is worth noting that the total gain of the full model exceeds the sum of individual module gains (2.3% + 2.9% + 2.6% = 7.8%). This nonlinear performance increment proves that the three modules are not simply superimposed but form stronger feature learning and detection capability through organic synergy. LMA is responsible for input quality improvement, DMC-Focus is responsible for feature discrimination enhancement, and DPFD is responsible for training efficiency boosting. The three modules cooperate layer by layer to achieve the overall performance leap, which fully verifies the rationality of the overall architecture design.

Apart from detection accuracy reflected by mAP@0.5, the parameter count and computational complexity are critical metrics for lightweight models oriented toward edge deployment. All parameter and GFLOPs statistics in this work are calculated using the thop package (Torch HOOK OP) [46], a widely adopted open-source tool for model complexity profiling in PyTorch. Figure 12 reports the number of model parameters (Params, unit: M) under different module combinations, and Figure 13 shows the corresponding computational complexity measured in GFLOPs. It is worth noting that DPFD is only activated during the training phase; the parameters and computation of the distillation branch are completely discarded in the inference stage, so the group “+DPFD” keeps the same Params and GFLOPs as the baseline model.

As shown in Figure 12, the baseline model possesses 2.84 M parameters. After introducing the LMA module, the parameter count rises slightly to 2.95 M, and the DMC module further increases parameters to 3.09 M. When LMA and DMC are both integrated, the parameter count reaches 3.20 M, which remains at a low lightweight level. Similarly, in terms of computational overhead in Figure 13, the baseline achieves 8.70 GFLOPs. Adding LMA increases GFLOPs to 9.20, and DMC brings it up to 9.90. For our full model with all modules, the total GFLOPs only reaches 10.5. Compared with the baseline, the final complete model brings limited increments in parameters and computation. Since DPFD branches are removed for inference, all additional overhead originates from LMA and DMC-Focus modules. Although these two modules introduce slight computational overhead, they bring prominent gains in crack detection accuracy, achieving a favorable trade-off between detection performance and the model’s lightweight property.

To further dissect the effectiveness of each sub-branch inside the DPFD strategy, we perform an internal ablation analysis on the DPFD module based on the complete LDD-YOLOv11 model. As shown in Figure 14, we remove the feature distillation branch, response distillation branch, and prototype contrast branch and record the corresponding mAP@0.5 changes.

When the feature distillation branch is removed (w/o feature distillation), mAP@0.5 drops from 90.5% to 88.2%, with a decrease of 2.3 percentage points. This indicates that feature-level knowledge transfer from the teacher model plays a core role in enhancing the fine-grained feature extraction ability of the student network, especially for the weak texture features of tiny cracks.

Removing the response distillation branch (w/o response distillation) leads to an mAP@0.5 of 87.9%, corresponding to a 2.6 percentage point performance loss. This result demonstrates that response distillation transfers the output distribution prior of the teacher model, which effectively improves the calibration of detection confidence and the generalization ability on cross-scene samples.

When only the prototype contrast branch is removed (w/o prototype contrast), mAP@0.5 decreases to 88.5%, with a reduction of 2.0 percentage points. This verifies that the prototype contrast mechanism helps enlarge the inter-class feature distance between crack targets and background clutter, thus reducing false positive detections.

When all three DPFD branches are removed (w/o all DPFD), the model performance drops to 85.9%, which is consistent with the +LMA + DMC combination in the previous module-level experiments. The total performance gain brought by the complete DPFD strategy reaches 4.6 percentage points. The performance degradation of removing every single branch is smaller than the total gain of the complete DPFD, indicating that the three distillation branches have complementary effects and jointly promote the performance improvement.

The internal ablation results confirm that all three sub-branches of DPFD make positive contributions to the model performance. The multi-branch distillation design realizes knowledge transfer from multiple dimensions, including feature level, response level, and prototype level, which effectively boosts the detection accuracy of the lightweight student model.

#### 4.3.2. Comparative Studies

To comprehensively validate the performance of the proposed LDD-YOLOv11 framework, we conduct comparative experiments with two categories of mainstream crack detection methods on the self-built building crack dataset: object detection models and semantic segmentation-based methods. The evaluation covers three dimensions—detection accuracy, model parameter scale, and computational complexity—to demonstrate the comprehensive advantages of the proposed method in both detection precision and lightweight deployability.

The comparison baselines are selected following three principles: representativeness of mainstream deployment architectures, coverage of different technical paradigms, and alignment with lightweight edge application scenarios. Specifically, we have the following:

1. Lightweight YOLO series detectors (YOLOv5n [47], YOLOv8n [48], YOLOv9t [49], and YOLOv11n [50]) are selected as the representative anchor-based detection baselines. As the most widely adopted architectures for real-time edge inspection, these models cover multiple generations of lightweight optimization iterations and reflect the current mainstream level of engineering-oriented lightweight detection algorithms. Comparing with these models can directly verify the performance improvement of the proposed method relative to common industrial solutions.
2. RT-DETR-L [51] is included as the representative transformer-based detection model. It serves as a typical baseline of the query-based detection paradigm, which helps compare the adaptability of anchor-based YOLO architectures and transformer architectures for slender crack targets under lightweight constraints.
3. Semantic segmentation methods including FPHB [52], DeepCrack [53], U-Net [25], and CrackFormer-II [54] are selected as the cross-paradigm comparison group. As the traditional mainstream technical route for crack detection, pixel-level segmentation methods are still widely used in engineering inspection scenarios. The comparison with segmentation methods can verify the engineering application value of the proposed detection framework.

Figure 15 illustrates the mAP@0.5 performance of different object detection models, covering classic lightweight YOLO series detectors and the transformer-based RT-DETR-L. As can be observed, the proposed LDD-YOLOv11 achieves the highest mAP@0.5 of 90.5%, outperforming all compared baseline models.

Specifically, compared with the YOLOv11n baseline, which obtains 88.3% mAP@0.5, the proposed method brings an absolute improvement of 2.2 percentage points. This result confirms that the embedded LMA module, DMC-Focus module, and DPFD strategy can effectively enhance the extraction and discrimination of fine crack features, thus improving the overall detection accuracy. In addition, LDD-YOLOv11 also surpasses other lightweight YOLO variants, including YOLOv8n (88.3%), YOLOv5n (79.9%), and YOLOv9t (79.8%), by distinct margins. It is notable that RT-DETR-L, despite its much larger model capacity, only achieves 65.2% mAP@0.5 on this fine crack detection task. This indicates that anchor-based YOLO architectures are more adaptable to the characteristics of slender crack targets than transformer-based detection paradigms under lightweight constraints.

For cross-paradigm comparison with semantic segmentation methods, we follow the unified evaluation protocol described in Section 4.1.3: Pixel-level segmentation outputs are converted into bounding boxes via minimum bounding rectangle extraction, and then evaluated using the same box-level metrics. The F1-score and IoU results are presented in Figure 16. LDD-YOLOv11 achieves 87.4% on box-level F1 scores (consistent with the precision of 91.6% and recall of 83.5% reported in Table 3), which is significantly higher than all segmentation-based methods. After conversion to bounding boxes, FPHB, DeepCrack, U-Net, and CrackFormer-II only obtain F1 scores of 25.8%, 24%, 20.1%, and 14.4%, respectively. The performance gap mainly comes from the fact that semantic segmentation models are optimized for per-pixel classification rather than bounding box regression, so the converted bounding boxes usually have deviations in position and scale from the ground truth. The results demonstrate that the proposed detection framework has inherent advantages in accurate crack localization, which is more compatible with the regional defect location requirements in practical engineering inspection.

To further evaluate the deployment potential on edge platforms, we compare the parameter quantity (Params) and floating-point operations (GFLOPs) of all models, as shown in Figure 17 and Figure 18.

In terms of parameter scale, LDD-YOLOv11 contains 3.2 M parameters, maintaining the same lightweight magnitude as mainstream YOLO lightweight models. Compared with the YOLOv11n baseline (2.84 M), the parameter increment is only about 12.7%, which is much lower than the 2.2 percentage point improvement in mAP@0.5. This means that the performance improvement is achieved at a very small cost of parameter overhead. As a reference, RT-DETR-L has as many as 32 M parameters, which is 10 times the scale of LDD-YOLOv11, but its detection accuracy is far inferior.

For computational complexity, LDD-YOLOv11 requires 10.5 GFLOPs for a single 640 × 640 image inference. Compared with YOLOv11n (8.7 GFLOPs), the computational cost increases by approximately 20.7%, which remains acceptable for real-time inference on resource-constrained edge devices. In contrast, RT-DETR-L requires up to 92 GFLOPs, nearly 9 times the computational overhead of our method.

Overall, the proposed LDD-YOLOv11 achieves a state-of-the-art accuracy–efficiency trade-off. It obtains the highest detection accuracy while retaining ultra-lightweight parameters and low computational complexity, which fully satisfies the deployment requirements of mobile unmanned inspection platforms.

Then, the comprehensive performance comparison results are presented in the radar chart (Figure 19), and the corresponding quantitative data are summarized in Table 3. The proposed LDD-YOLOv11 is compared with seven representative methods across five core dimensions: mAP@0.5, precision (P), recall (R), inference frame rate (FPS), and model parameter count (Params). It should be noted that all FPS metrics in this comparison are measured on an NVIDIA RTX 4090 desktop GPU under a unified single-frame end-to-end inference protocol to ensure fair cross-model benchmarking. These values differ from the general-purpose CPU inference latency and Jetson batch throughput reported in the subsequent edge deployment verification section.

The comparison baseline covers multiple generations of lightweight YOLO detectors, which helps verify that the proposed method achieves better fine crack detection under lightweight constraints without excessive parameter growth. The baseline also includes the transformer-based RT-DETR-L, as well as classical crack segmentation methods including U-Net and DeepCrack.

For cross-paradigm comparison with segmentation methods, we select U-Net and DeepCrack as representative baselines for two reasons. First, U-Net is the most classic and widely recognized general semantic segmentation architecture, which has served as the foundational baseline for numerous crack segmentation studies; DeepCrack is a classical dedicated crack segmentation method that is specifically optimized for crack features. These two methods, respectively, represent the general-purpose segmentation paradigm and the task-specific crack segmentation paradigm, which can fully reflect the performance level of the segmentation-based technical route on crack detection tasks. Second, since the core difference between detection and segmentation paradigms lies in the output form rather than intra-paradigm differences, selecting two highly representative benchmarks is sufficient to support cross-paradigm performance comparison.

In terms of detection accuracy, LDD-YOLOv11 achieves the highest mAP@0.5 of 90.50% among all compared methods, which is 2.2 percentage points higher than the YOLOv11n baseline. It also substantially outperforms other lightweight variants, including YOLOv8n, YOLOv5n, and YOLOv9t and presents a dominant advantage over RT-DETR-L, U-Net, and DeepCrack. For the core detection metrics, LDD-YOLOv11 reaches 91.6% precision and 83.5% recall, both ranking first among all object detection models. This result demonstrates that the proposed LMA and DMC-Focus modules effectively improve the model’s capability to accurately locate crack targets and simultaneously reduce missed detection and false positive rates.

In terms of inference efficiency and lightweight property, LDD-YOLOv11 delivers an inference speed of 67.7 FPS with only 3.20 M parameters. Due to the introduction of feature enhancement modules, its FPS is slightly lower than that of YOLOv11n (92.9 FPS) and YOLOv8n (121.9 FPS), but it still maintains sufficient real-time detection capability for engineering inspection scenarios. Meanwhile, the parameter count of 3.20 M remains at an excellent lightweight level, which is approximately one tenth of RT-DETR-L (32.0 M), less than half of U-Net (7.76 M), and far lower than DeepCrack (14.20 M).

It should be specially explained that U-Net and DeepCrack are typical semantic segmentation-based crack detection approaches. They generate pixel-level segmentation masks as output rather than the bounding box predictions adopted in object detection paradigms. Since precision and recall are calculated based on bounding box matching between predictions and ground truth, these two metrics cannot be directly derived for segmentation methods. Therefore, in this work, only mAP@0.5 is used for cross-paradigm performance comparison between segmentation methods and detection methods.

Overall, the proposed LDD-YOLOv11 exhibits remarkable comprehensive advantages. It achieves the optimal trade-off among detection accuracy, inference efficiency, and lightweight design. Specifically, it surpasses all lightweight detection baselines in accuracy, while maintaining much lower computational and parameter overhead than large-scale models and segmentation-based methods. This fully verifies the effectiveness and superiority of the proposed network architecture and optimization strategy in improving the comprehensive performance of building crack detection, and provides an efficient technical solution for crack detection tasks that require a balance between detection accuracy and real-time performance in practical engineering applications.

#### 4.3.3. Cross-Dataset Generalization

To evaluate the cross-domain generalization capability of the proposed model, we introduce the public CrackForest dataset (abbreviated as CFD in this work) as the out-of-domain test benchmark [55]. Released together with the classical random structured forest crack detection method, this dataset contains urban road pavement images with pixel-level ground truth annotations. It is one of the most widely adopted standard benchmarks for cross-scene crack detection generalization evaluation in the field.

For this cross-dataset generalization comparison, we uniformly adopt mAP@0.5 as the core evaluation metric for two reasons. First, considering the cross-paradigm comparison between anchor-based detection models and semantic segmentation models, precision and recall are calculated based on strict bounding box matching. For segmentation methods for which their native output is pixel-level masks, the converted minimum bounding rectangles usually have large deviations in position and scale, which makes precision and recall highly unstable and unable to fairly reflect the actual detection capability. As a comprehensive ranking metric, mAP@0.5 has higher tolerance for bounding box offsets and is more suitable for cross-paradigm horizontal comparison. Second, the core goal of this experiment is to quantify the overall performance degradation of models when transferring from the in-house building dataset to the out-of-domain road dataset. As a comprehensive accuracy indicator, mAP@0.5 is sufficient to reflect the generalization capability of models across domains, and further disaggregation into precision and recall is not necessary for generalization evaluation.

Figure 20 compares the mAP@0.5 performance of different detection models on the in-house source dataset and the public CFD dataset to evaluate the cross-dataset generalization capability. In this bar chart, the vertical axis denotes mAP@0.5 in percentage (%). The dark-blue bars represent results tested on our in-house building-crack dataset, and the red bars correspond to results obtained on the public CFD dataset. On our in-house dataset, the proposed LDD-YOLOv11 achieves the highest mAP@0.5 of 90.5%, outperforming YOLOv11n (88.30%), YOLOv8n (88.3%), and other baseline lightweight detectors. When transferred to the public CFD dataset with different crack distributions, background styles, and shooting conditions, all models suffer an obvious accuracy drop, which reveals the domain gap between real-world building crack data and public crack datasets. Even so, LDD-YOLOv11 still obtains 45.7% mAP@0.5 on the CFD dataset, which is competitive among all lightweight models and slightly higher than YOLOv9t (45.62%). The above results demonstrate that the LMA and DMC-Focus modules enhance the model’s capability to extract crack-oriented visual features. Combined with the constraint of DPFD prototype contrast distillation, the model maintains acceptable generalization performance toward out-of-domain crack samples. Nevertheless, there remains considerable room for further improvement for cross-domain adaptation, which will be regarded as one of our future research directions.

#### 4.3.4. Sensitivity Analysis of DPFD Hyperparameters

To verify the rationality of the default weight coefficients in the proposed DPFD strategy and evaluate the sensitivity of model performance to hyperparameter changes, we conduct single-factor controlled variable experiments on the validation split of the training dataset. All other training configurations and model structures are fixed, and typical values of the feature distillation weight α, response distillation weight β, and prototype contrast weight γ are traversed, respectively. The variation curves of mAP@0.5 on the validation set with each hyperparameter are shown in Figure 21. It should be noted that the mAP values here are obtained on the validation set for hyperparameter tuning, which differ from the final test-set mAP@0.5 of 90.5% reported in previous sections.

As presented in Figure 21a, the detection accuracy first increases and then decreases with the rise of α, and it reaches the peak mAP@0.5 of 0.8053 when α = 1.0. When α is lower than 1.0, the deep feature knowledge transferred from the teacher model is insufficient, so the enhancement effect on weak fine crack features is limited. When α exceeds 1.0, excessively strong feature-level distillation will constrain the independent feature learning ability of the lightweight student model and lead to performance degradation instead.

For the response distillation weight β shown in Figure 21b, the model also achieves the optimal mAP@0.5 of 0.8053 at β = 0.5. A smaller β cannot fully exploit the output distribution prior knowledge of the teacher model. In contrast, an overlarge β will cause the student model to overfit the response pattern of the teacher model and damage its generalization ability on unseen building crack samples.

Figure 21c illustrates the influence of the prototype contrast weight γ. The optimal value is 0.3, which achieves the best mAP@0.5 of 0.8053. The prototype contrast loss is designed to enhance the feature discriminability between crack prototypes and background prototypes. When γ is too low, the inter-class feature distance constraint is insufficient. When γ is higher than 0.3, performance decreases slowly rather than dropping sharply, indicating that excessive prototype contrast will introduce additional optimization noise and slightly interfere with the main detection task.

Overall, the mAP@0.5 of the model maintains a small fluctuation range near the optimal values of the three hyperparameters. The result demonstrates that the proposed model has good robustness to hyperparameter adjustment, and the performance improvement does not rely on finely tuned hyperparameters. The default setting of α = 1.0, β = 0.5, and γ = 0.3 achieves a reasonable balance among feature distillation, response distillation and prototype contrast, which is adopted in all subsequent experiments.

#### 4.3.5. Edge Deployment Verification

Figure 22 shows the end-to-end inference latency and frame rate of different models tested on a general-purpose CPU, which simulates the low-computing-power condition for practical deployment. In this figure, the left vertical axis corresponds to end-to-end single-frame inference latency in milliseconds (ms), and the right vertical axis represents the inference frame rate (FPS). The bar chart denotes end-to-end inference latency, while the line chart reflects the corresponding frame rate. As can be observed, YOLOv11-tiny achieves the optimal speed performance with an end-to-end latency of 29.7 ms and a frame rate of 33.7 FPS. Benefiting from the lightweight backbone, YOLOv11n and YOLOv8n obtain comparable inference performance, reaching 19.1 FPS and 18.7 FPS, respectively. Our proposed LDD-YOLOv11 introduces the LMA, DMC-Focus, and DPFD optimization modules, which result in extra computational overhead. Its end-to-end latency rises to 68.4 ms with 14.6 FPS on CPU. It should be noted that all distillation-related branches are completely removed during inference, and the additional latency is mainly contributed by the LMA and DMC-Focus modules. Although the CPU-side speed is inferior to the baseline tiny/nano versions, it still maintains stable inference capability for crack inspection tasks.

Further, Figure 23 evaluates the simulated peak batch throughput of LDD-YOLOv11 on the Jetson Orin NX embedded platform under different computing-efficiency constraints. It should be clarified that the throughput values here represent the theoretical maximum continuous inference capability under batch processing with TensorRT optimization rather than the end-to-end single-frame inference frame rate reported in previous CPU and desktop GPU tests. Specifically, the single-frame end-to-end inference speed of the model is 67.7 FPS on the desktop RTX 4090 GPU and 14.6 FPS on a general-purpose CPU, while the batch throughput metric reflects the upper limit of processing capacity when the device runs at full load with continuous image streams.

In this bar chart, the horizontal axis denotes computing-efficiency constraints (100%, 50%, and 30%), which simulate practical working conditions such as hardware frequency reduction and multi-task load on edge devices. The vertical axis represents simulated inference throughput measured in FPS. The light-blue bars correspond to Jetson Orin NX (8 GB), while dark-green bars represent Jetson Orin NX (16 GB). Under full computing efficiency (100%), the simulated peak throughput reaches 3333 FPS for the 8 GB version and 4762 FPS for the 16 GB version. When the computing efficiency drops to 50% and 30%, the inference throughput decreases proportionally for both memory configurations. Even under the strict 30% computing-efficiency limitation, the model still maintains a throughput of 1000 FPS (8 GB) and 1429 FPS (16 GB).

Combined with the previous CPU-side test results, it can be concluded that when equipped with embedded GPU acceleration, LDD-YOLOv11 achieves extremely high inference throughput capacity and exhibits stable performance degradation under resource-limited scenarios. All distillation branches are discarded in the inference phase; only LMA and DMC-Focus modules participate in forward propagation. Considering that our model is mainly oriented to GPU-accelerated edge embedded devices rather than pure CPU operation, the above results demonstrate that the proposed model possesses satisfactory adaptability for real-world unmanned inspection edge-deployment scenarios.

## 5. Discussion

The proposed LDD-YOLOv11 achieves outstanding lightweight and high-precision detection performance for building cracks, and it exhibits strong adaptability to complicated on-site inspection environments. Nevertheless, combined with practical engineering demands and experimental outcomes, the current model still has several limitations that need to be optimized, which also provide clear directions for our follow-up research.

Firstly, in terms of crack recognition accuracy, the model still suffers from fluctuating recognition performance when dealing with ultra-fine cracks and cracks under extremely low-contrast conditions. On the one hand, the characteristic signals of such tiny cracks are extremely weak and easily masked by background clutter and illumination noise. On the other hand, limited by the lightweight network design, the shallow feature extraction layers have restricted receptive fields, and pixel-level crack features are prone to be lost during successive downsampling operations. As a result, the existing LMA and DMC-Focus modules still have room for improvement in capturing extremely weak crack features.

Secondly, in terms of crack localization accuracy, when the model performs bounding box regression on crack patterns, it cannot achieve completely precise localization for continuous long cracks. In some cases, a single continuous long crack is segmented into multiple separated detection boxes. This issue arises from two levels of causes. In terms of network mechanism, the one-stage anchor-free detection paradigm inherently lacks long-range continuous dependency modeling, so over-length linear targets tend to be split into multiple independent predictions. In terms of practical interference, the irregular shape of cracks, complex background disturbances, and the constraint effect of the regression loss function also aggravate the segmentation of detection results, which further affects the integrity and localization accuracy of crack detection.

Finally, in terms of long-term operational stability on edge platforms, during long-term continuous inspection missions of the unmanned mobile platform, problems such as gradual degradation of model inference speed and insufficient overall stability may occur. From the perspective of algorithm optimization, the current model has not been subjected to operator fusion and quantization optimization tailored for edge devices, so heat accumulation during continuous inference will cause frequency throttling of computing hardware and result in frame rate fluctuations. In addition, fluctuations in available computing power during the movement of the mobile platform, memory usage accumulation during long-term model operation, and continuous interference from environmental illumination and vibration also jointly affect operational stability.

Notably, the above analysis of operational stability is based on theoretical inference simulation and standalone hardware benchmark tests. The proposed algorithm has not yet been deployed and validated on a physical unmanned mobile inspection platform. The end-to-end performance of the full inspection workflow, including on-board image acquisition latency, platform vibration-induced motion blur, dynamic viewing angle variation, and on-board computing resource scheduling, still needs to be verified in actual field inspection scenarios.

In response to the above deficiencies, in the future, in-depth research will be focused on the following three directions to promote the transformation of this unmanned mobile platform from initial practical application to higher-level and more stable engineering application.

Firstly, a lightweight crack segmentation branch will be introduced to realize pixel-level crack detection and automatic width quantification. On the basis of retaining the existing lightweight detection backbone, a lightweight instance segmentation head will be added to generate pixel-level crack masks without significantly increasing parameter overhead. Combined with a skeleton extraction-based crack quantification algorithm, key geometric parameters such as crack length, average width, and maximum width can be calculated automatically, providing more accurate quantitative data support for structural damage assessment in engineering inspection.

Secondly, temporal information between consecutive video frames will be fused to suppress motion blur and improve inspection video detection performance. Aiming at jitter, motion blur, and perspective change during the movement of the inspection platform, an inter-frame feature alignment module and digital image stabilization preprocessing will be introduced. By leveraging the spatiotemporal consistency of crack targets in continuous video frames, the interference of motion blur and viewing angle distortion on feature extraction can be effectively reduced, ensuring that the model maintains stable and reliable detection performance in actual mobile inspection scenarios.

Thirdly, model quantization, pruning, and operator fusion optimization will be carried out to adapt to lower-power edge inspection terminals. Specifically, INT8 quantization, structured channel pruning, and convolutional operator fusion technologies will be applied to the framework to compress model volume, reduce memory occupation, and lower computing overhead. On this basis, the optimized model will be transplanted to a physical unmanned inspection platform, including both unmanned aerial vehicles and ground patrol robots, to construct a complete end-to-end inspection pipeline integrating on-site image acquisition, real-time inference, and structural defect feedback. These optimizations can alleviate heat accumulation and computing power fluctuation during long-term continuous inference, effectively improve inference speed and long-term operational stability on mobile platforms, and enable the model to be deployed on more low-power embedded inspection devices.

## 6. Conclusions

In this study, we propose a lightweight LDD-YOLOv11 model tailored for engineering crack detection in complex scenarios. Targeting the inherent limitations of conventional lightweight crack detection models, including insufficient feature extraction for blurry cracks under extreme illumination, poor adaptability to complex backgrounds, and weak cross-scene generalization, the proposed model achieves comprehensive optimization of the baseline YOLOv11 framework.

This paper takes the high-performance YOLOv11 as the basic baseline model and combines the actual pain points of crack detection to carry out multi-module collaborative optimization design.

Firstly, the introduction of the LMA module specifically addresses the issues of different lighting variations and material differences, effectively solving the problems of blurred crack features and being submerged by interfering factors under extreme lighting conditions. It significantly enhances the model’s crack recognition capabilities in complex lighting scenarios such as strong light, water stain reflection, and dark environments.

Secondly, the DMC-Focus module was designed. Through the dual mechanisms of direction constraints and reflection constraints, it accurately identifies gradient features, effectively distinguishes the real crack boundaries from the pseudo-boundaries generated by highlights and complex backgrounds, and further enhances the feature extraction and recognition accuracy of fine cracks.

Finally, by integrating DPFD, it not only achieves lightweight compression of the model and meets the deployment requirements of mobile platforms but also effectively enhances the accuracy and inference speed of crack detection in wide-domain scenarios, realizing the collaborative optimization of detection performance and lightweight deployment.

Comprehensive ablation and comparative experiments on crack datasets verify that the proposed LDD-YOLOv11 achieves superior mAP@0.5 (90.5%) and real-time inference speed (FPS = 67.7) with fewer parameters (3.20 M) compared with mainstream lightweight detection models, fully demonstrating the effectiveness of the three collaborative optimization modules.

However, this method exhibits limitations in the detection of ultra-fine cracks, box regression accuracy, and maintaining fast inference over extended periods. To further address these issues, future work will focus on integrating a pixel-level segmentation branch and quantitative computation modules. Future work will also seek to compress computational costs via parameter pruning and inference acceleration, along with incorporating image stabilization and viewpoint correction strategies to improve system robustness. We position LDD-YOLOv11 as a critical first step toward lightweight crack detection on unmanned platforms.

## Figures and Tables

**Figure 1:**
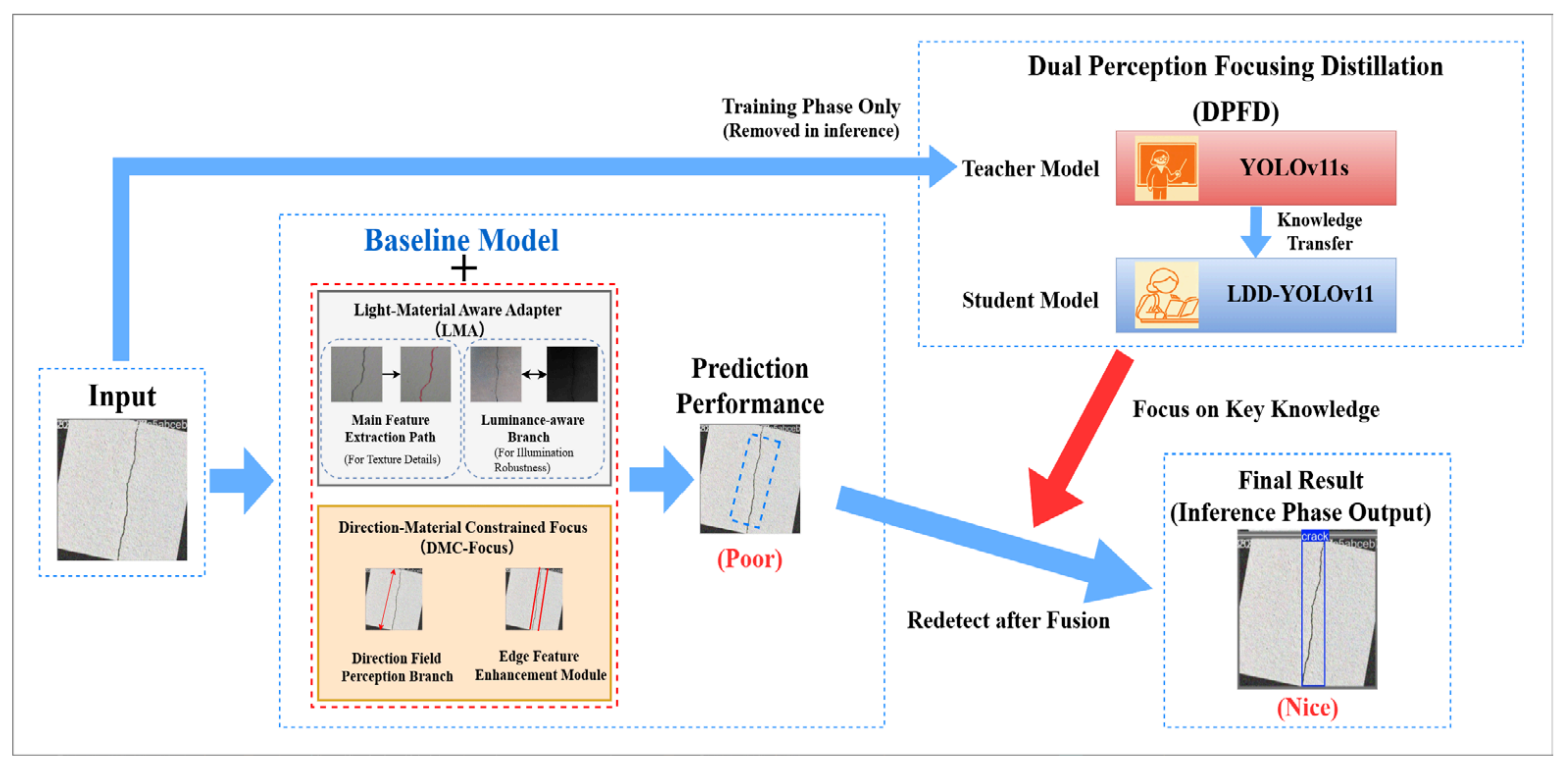
Overall framework of the proposed LDD-YOLOv11. The DPFD branch works only during the training phase and is completely removed during inference to ensure lightweight deployment.

**Figure 2:**
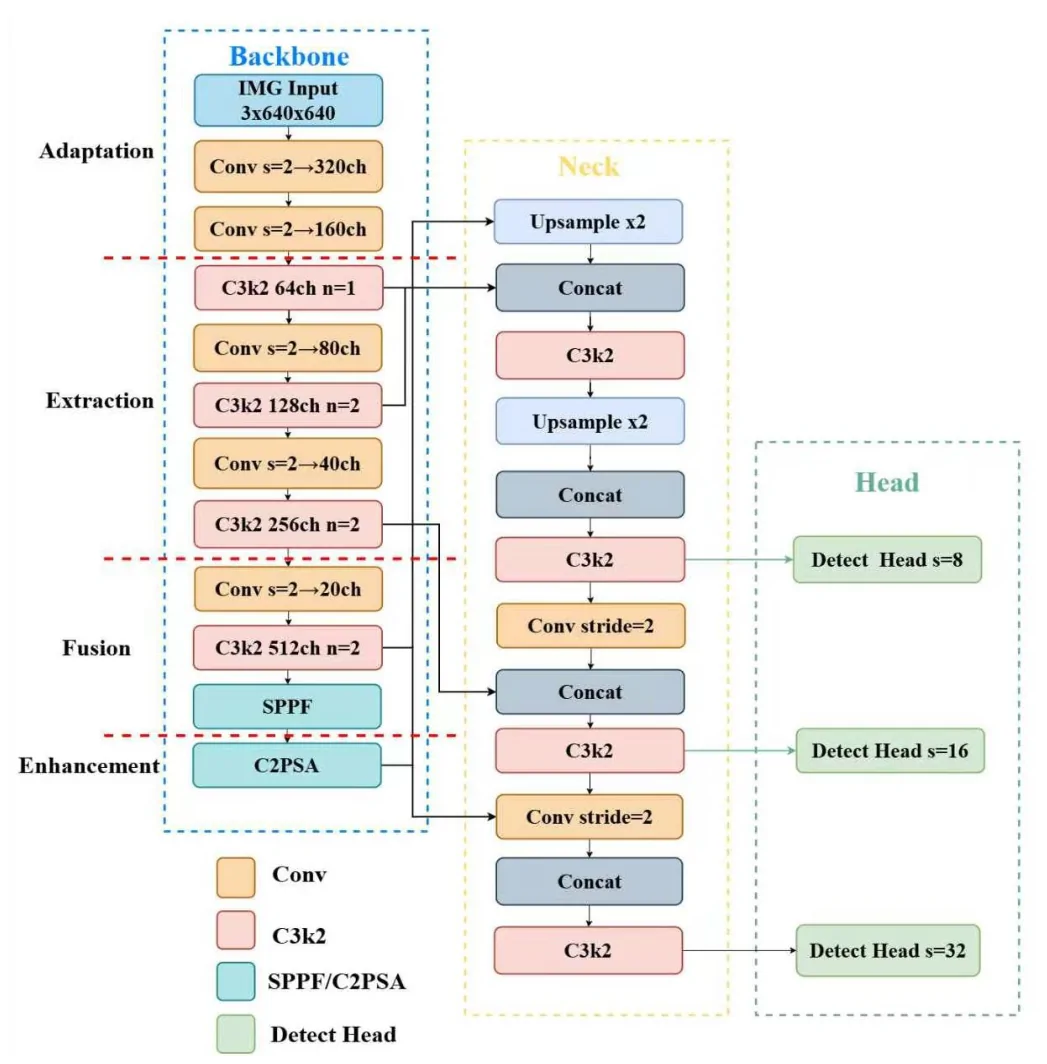
Original YOLOv11 network framework.

**Figure 3:**
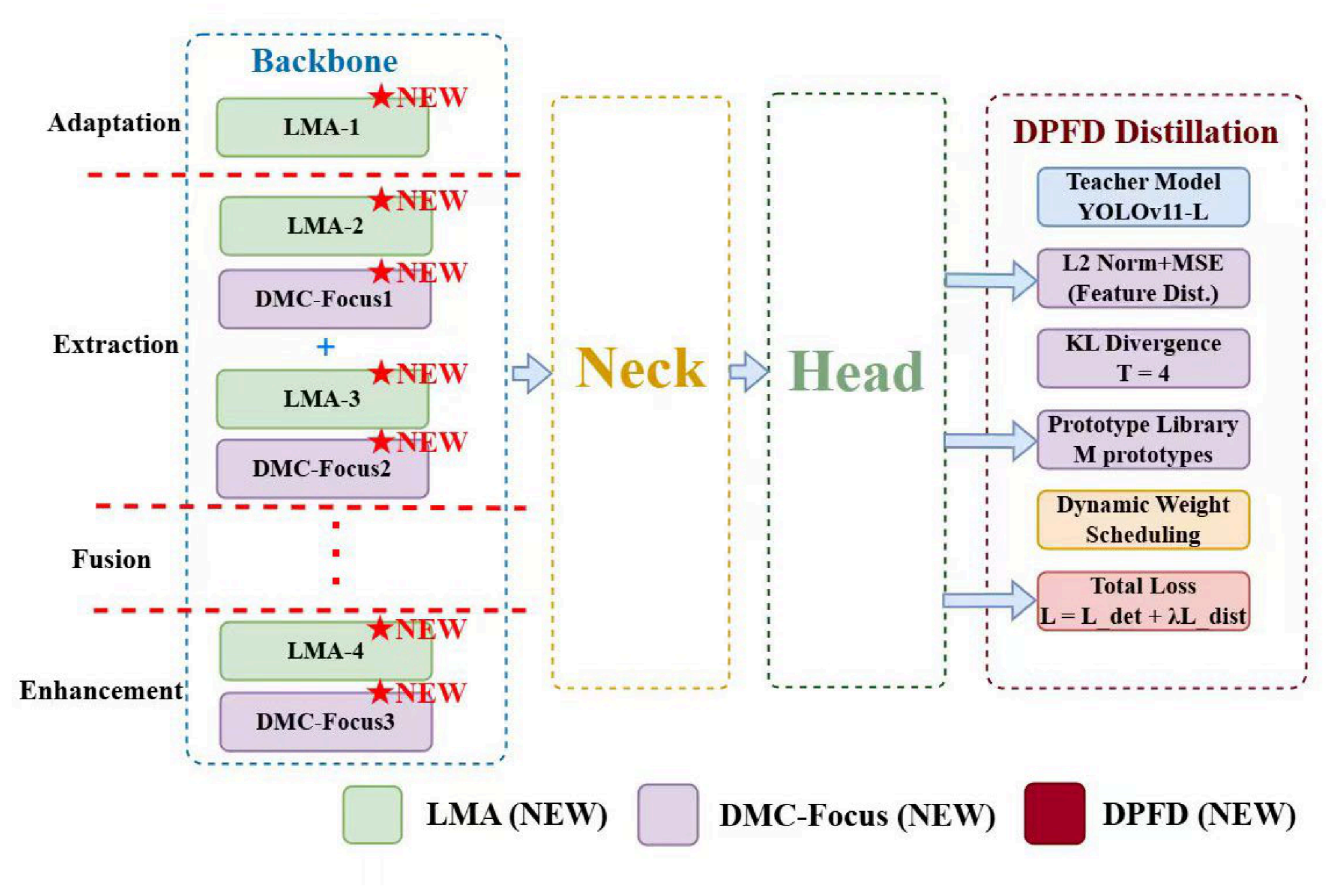
LDD-YOLOv11 network architecture.

**Figure 4:**
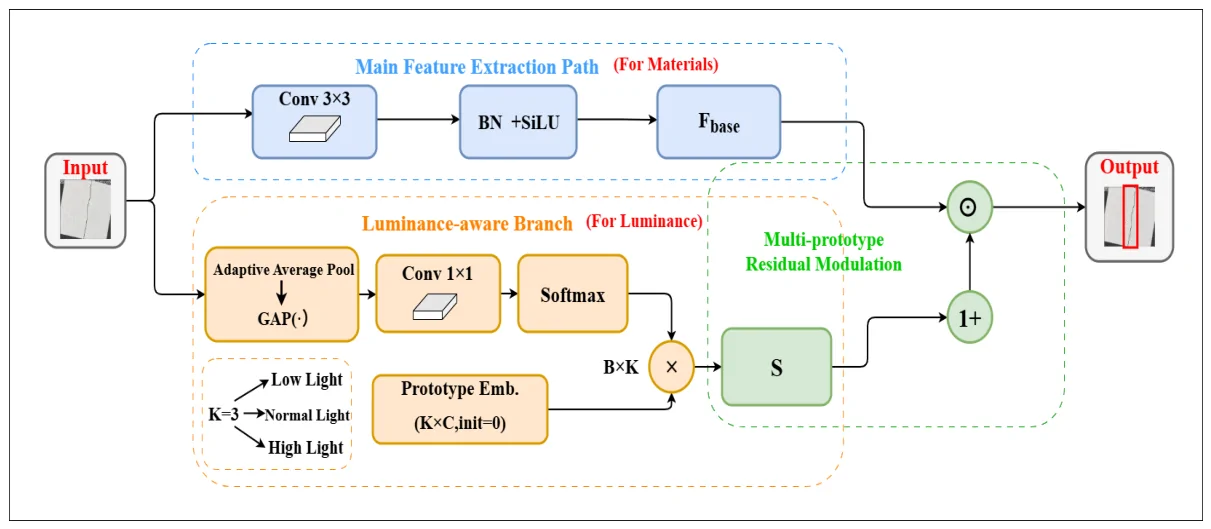
Illustration of the LMA module.

**Figure 5:**
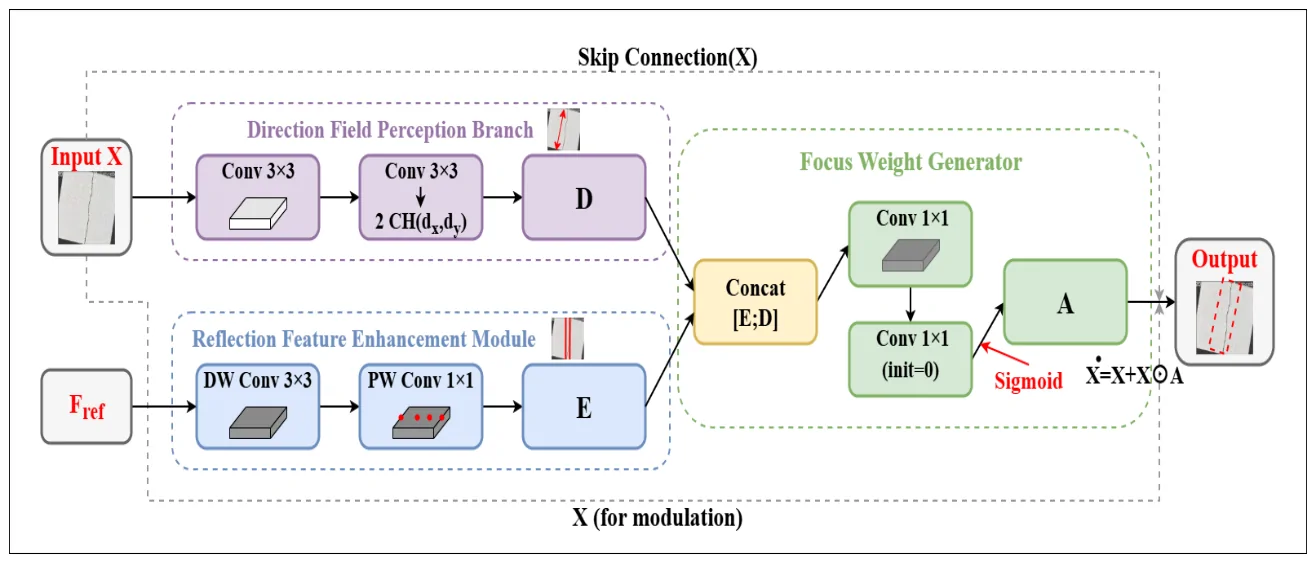
Illustration of the DMC-Focus module.

**Figure 6:**
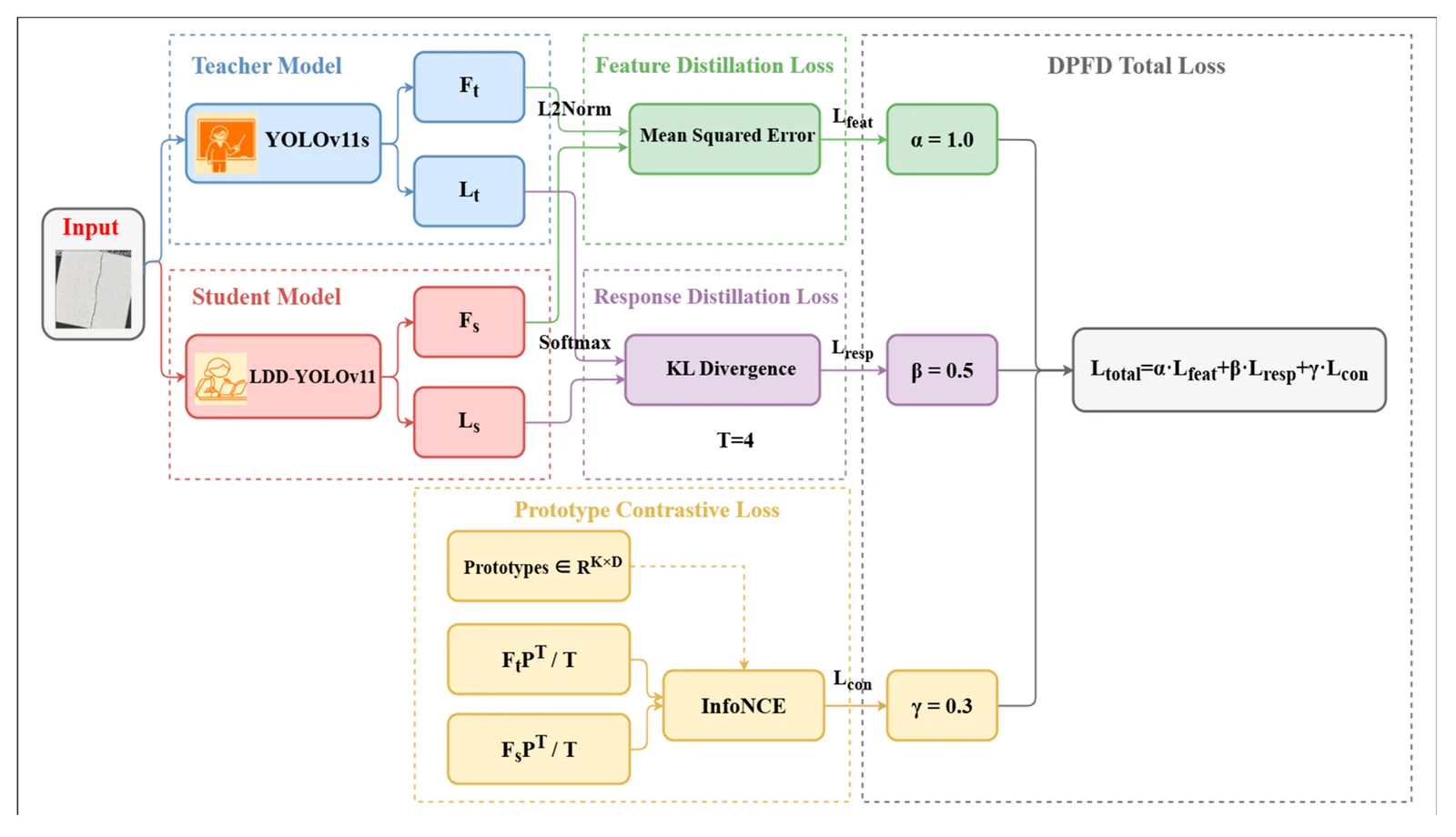
DPFD module framework.

**Figure 7:**
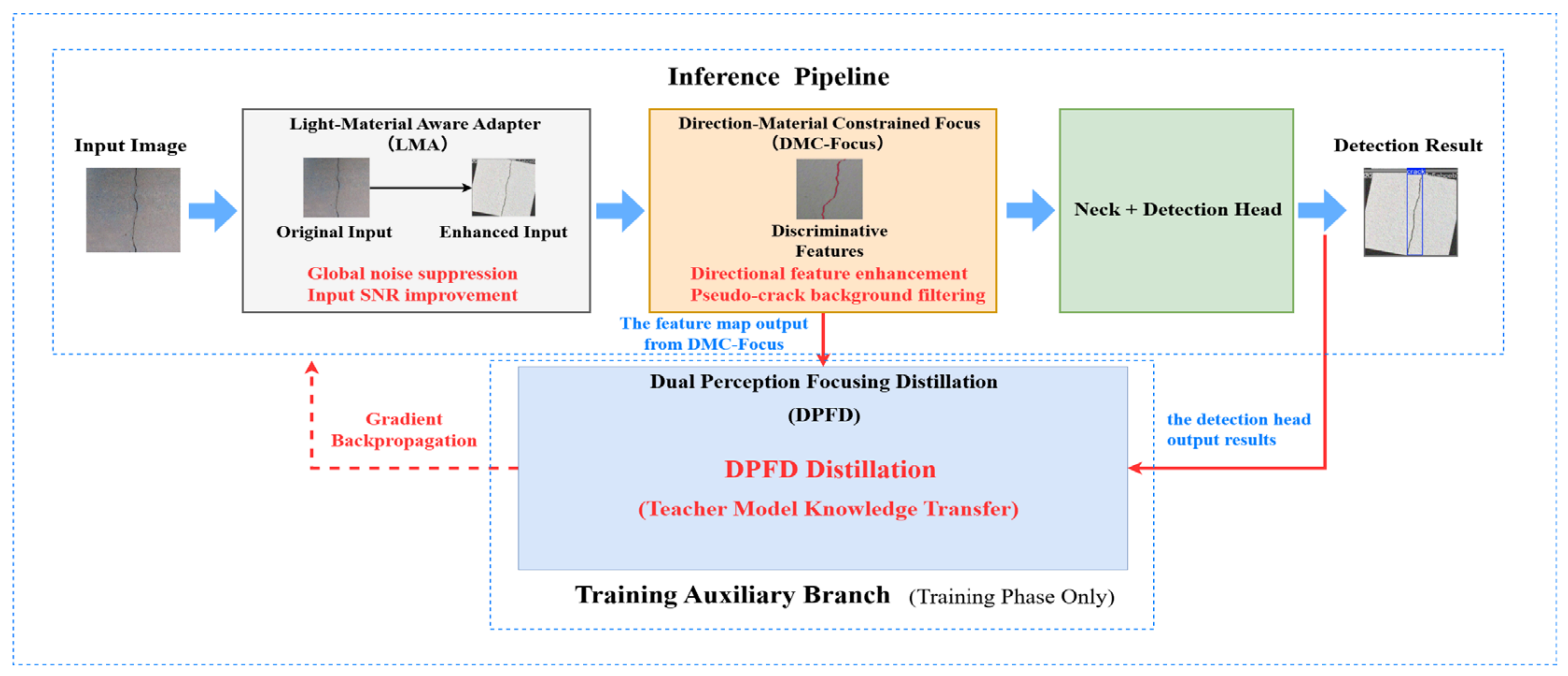
Illustration of the module synergistic mechanism and feature evolution process.

**Figure 8:**
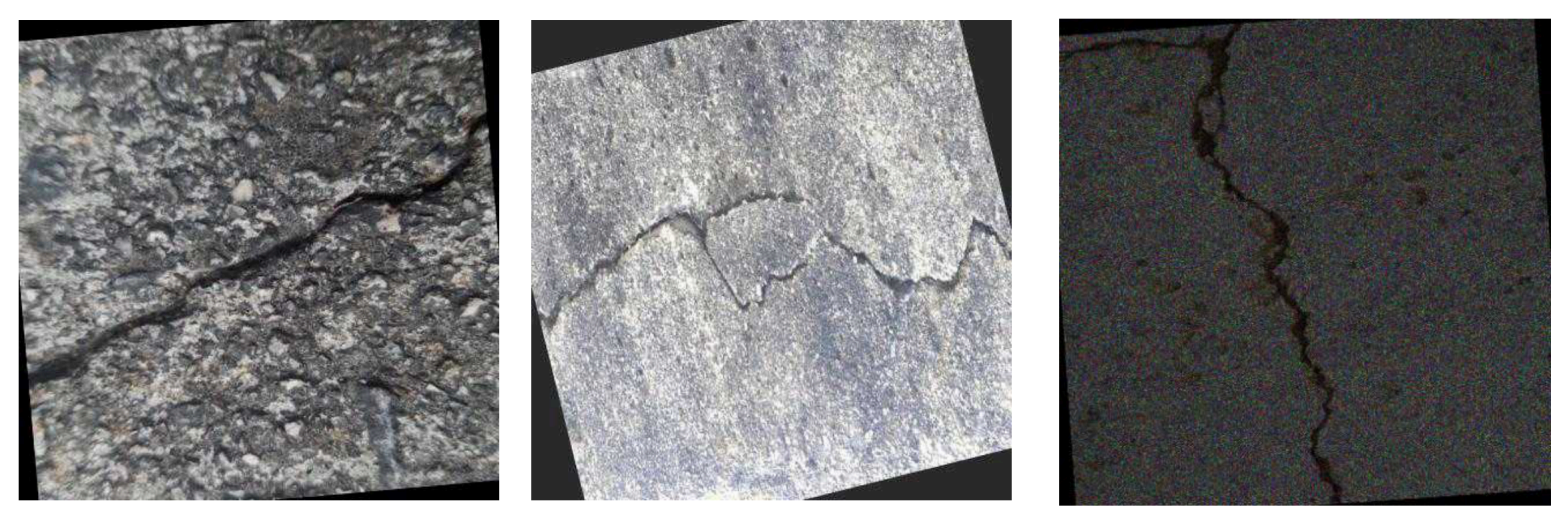
CCIC dataset.

**Figure 9:**
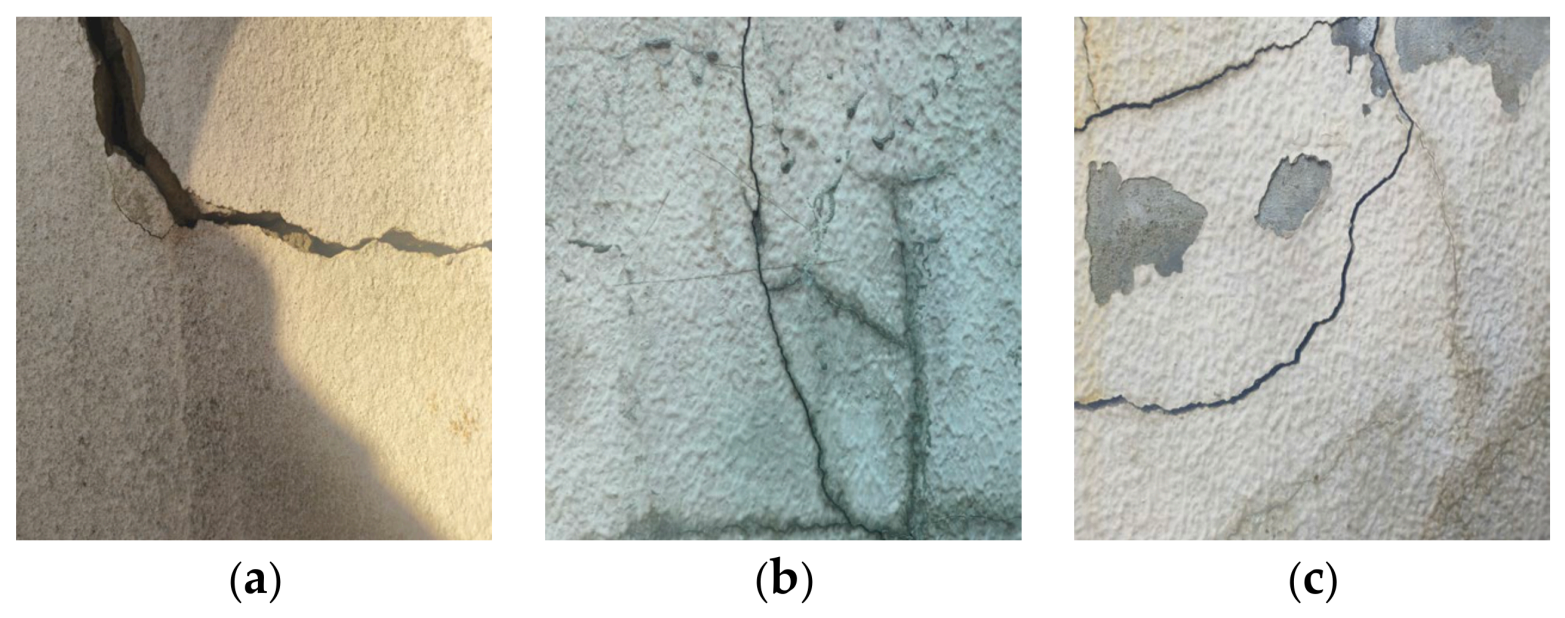
Self-built dataset: (**a**) wide cracks (>5 mm), strong light, masonry joints; (**b**) ultra-fine cracks (<1 mm), low light, water stains; (**c**) medium cracks (1–5 mm), normal light, stains/clutter.

**Figure 10:**
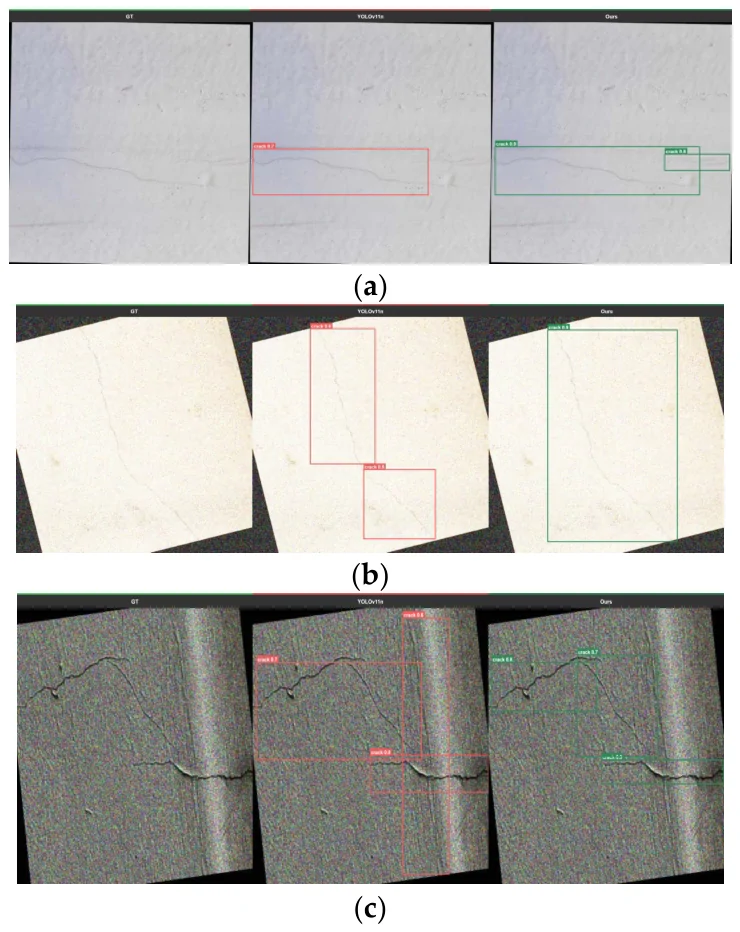
Model detection performance comparison. (**a**) Detection performance of some small-sized crack targets with high similarity to the background. (**b**) Detection performance of some crack targets with longer lengths. (**c**) Detection performance of multi-crack targets.

**Figure 11:**
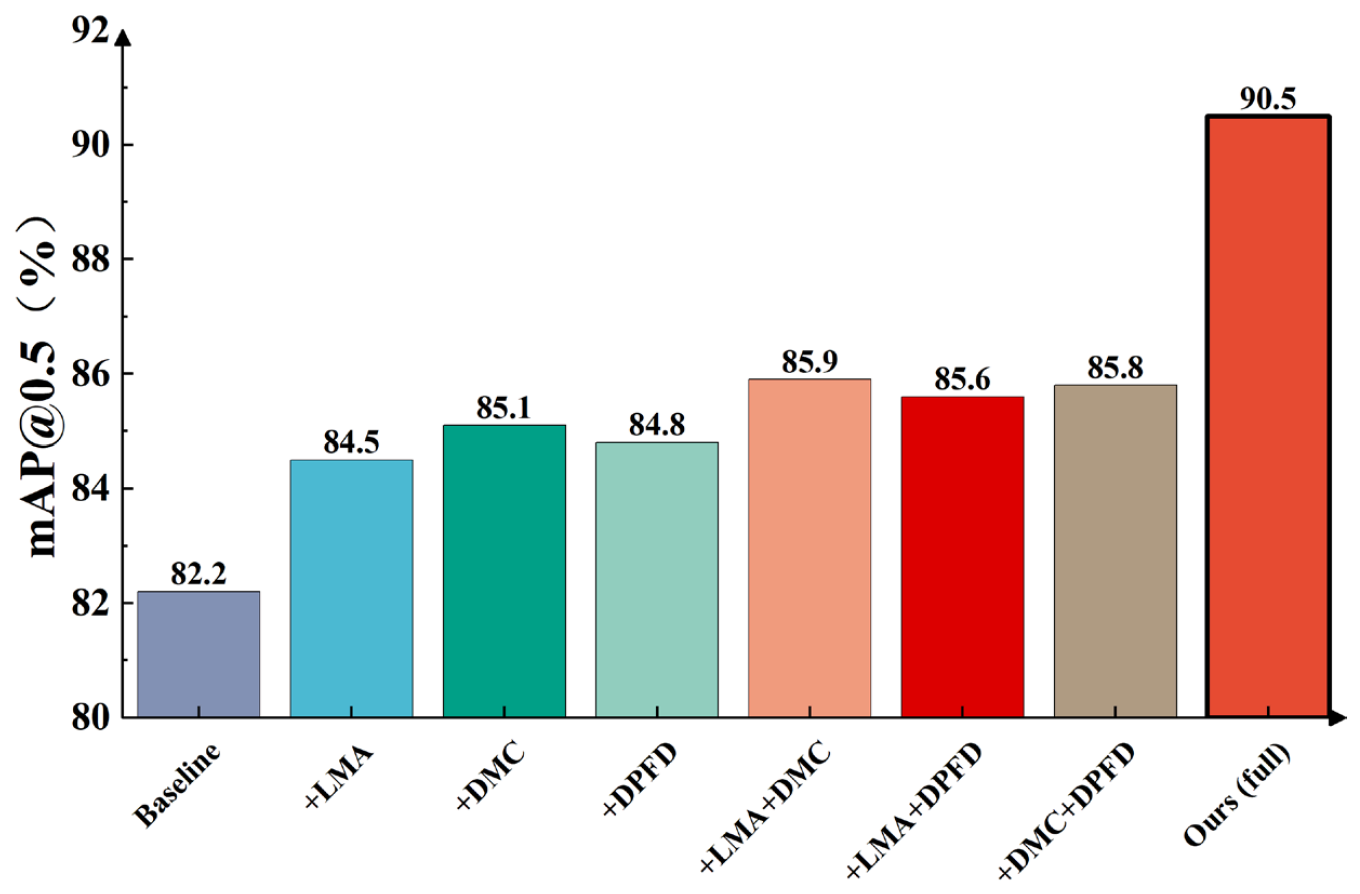
Comparative analysis of the mAP@0.5 metric after integrating combinations of different improvement modules.

**Figure 12:**
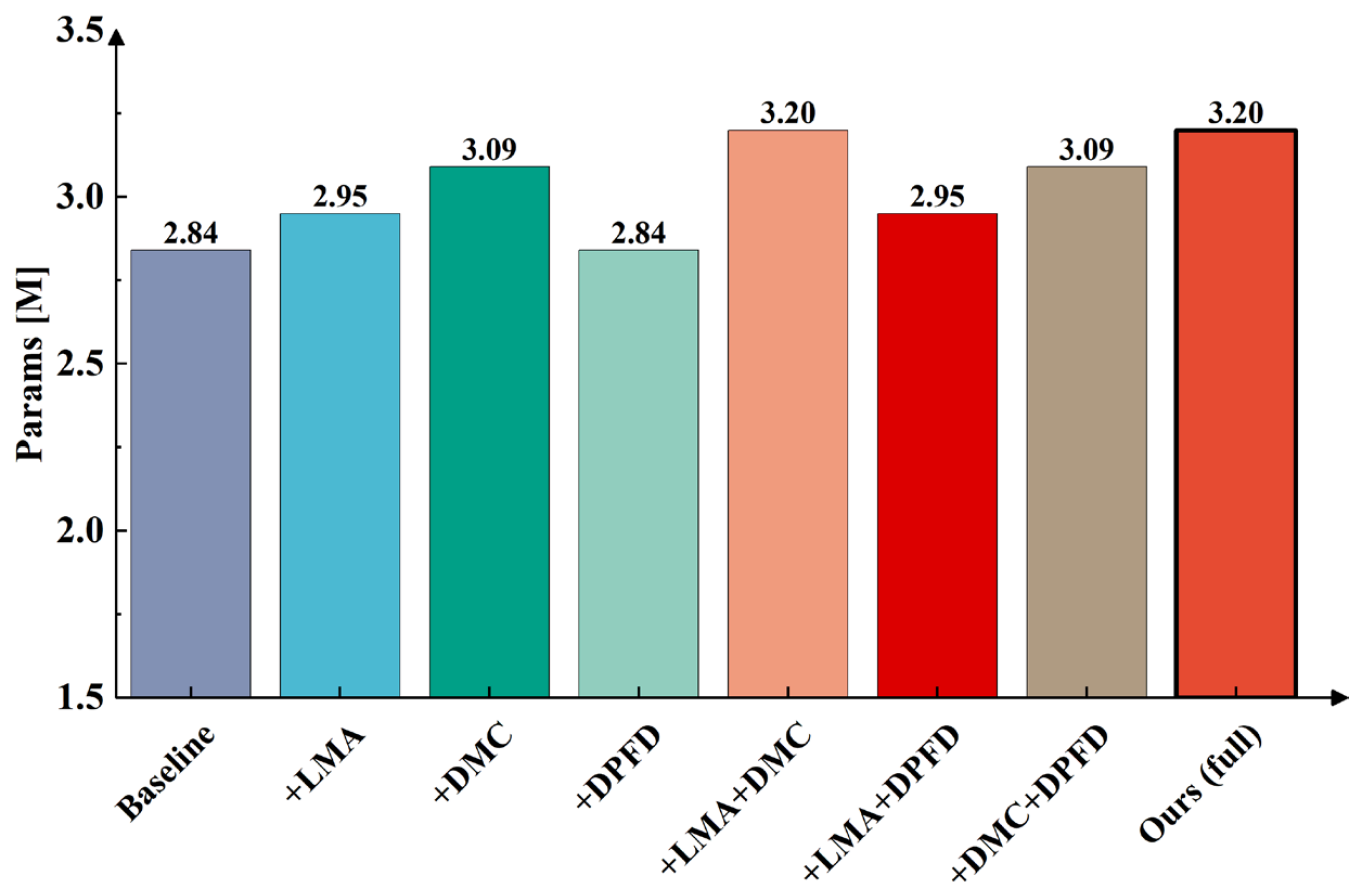
Parameter statistics under different ablation combinations.

**Figure 13:**
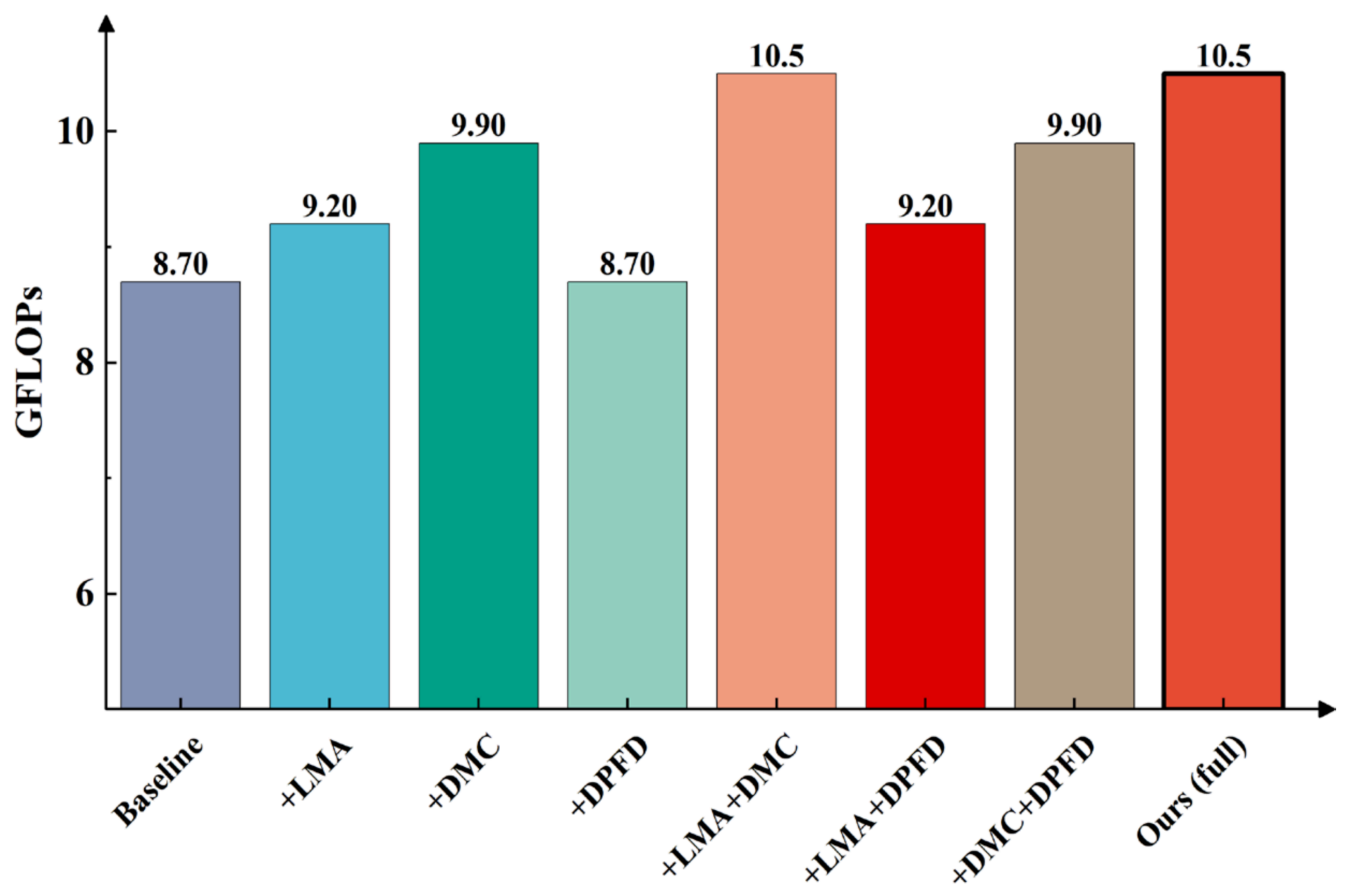
Computational complexity (GFLOPs) under different ablation combinations.

**Figure 14:**
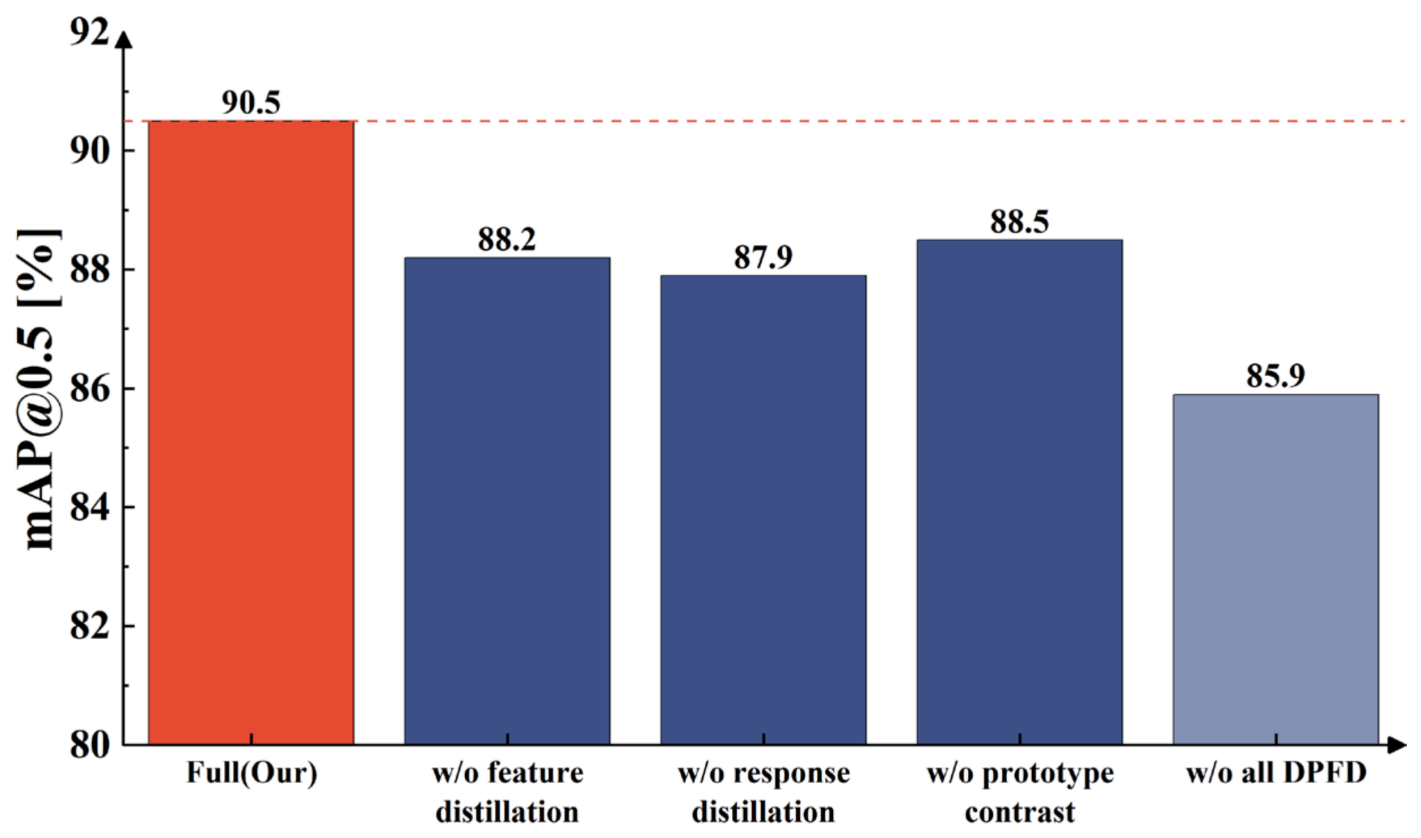
Internal ablation results of DPFD branches on mAP@0.5.

**Figure 15:**
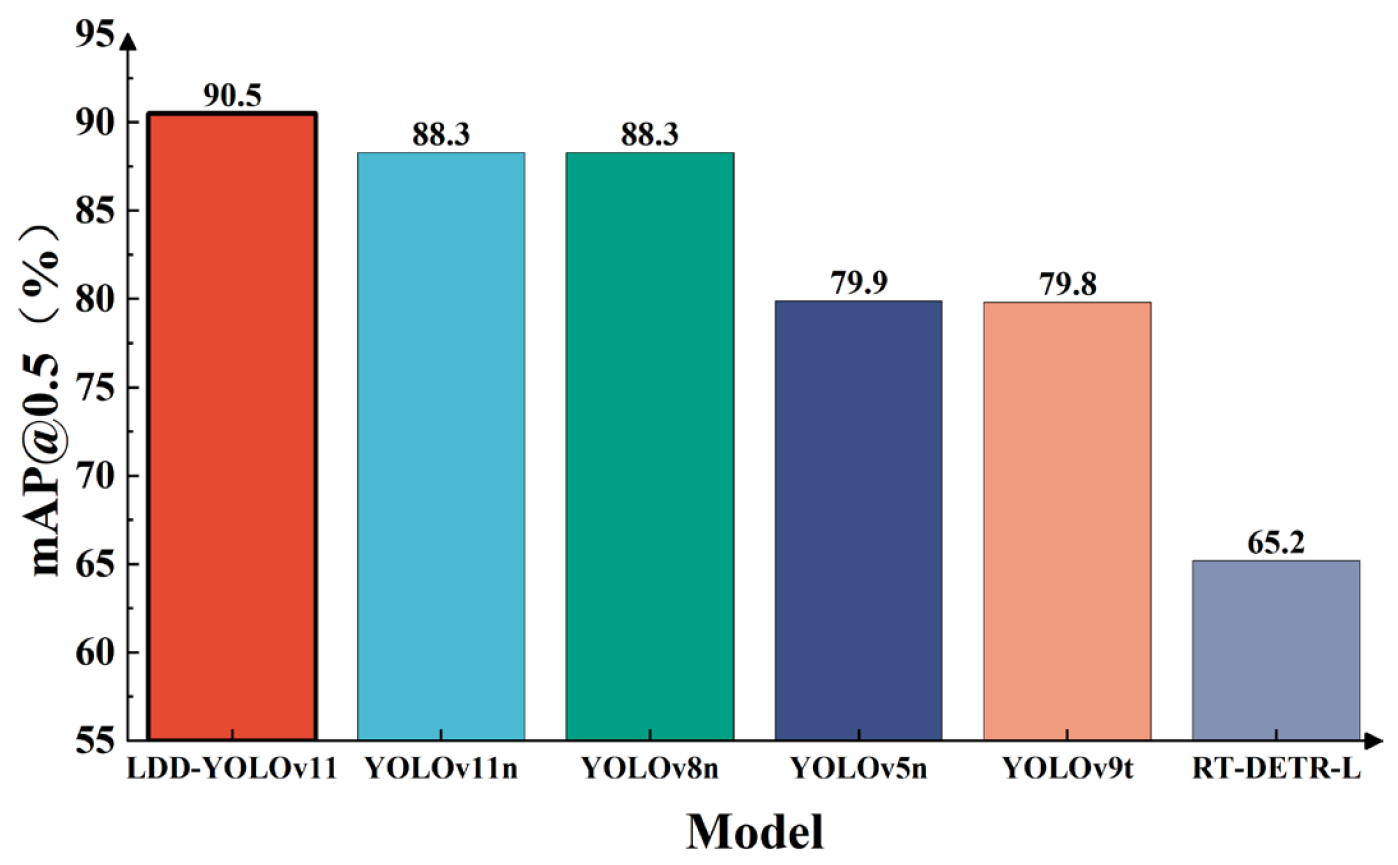
mAP@0.5 comparison of different object detection models.

**Figure 16:**
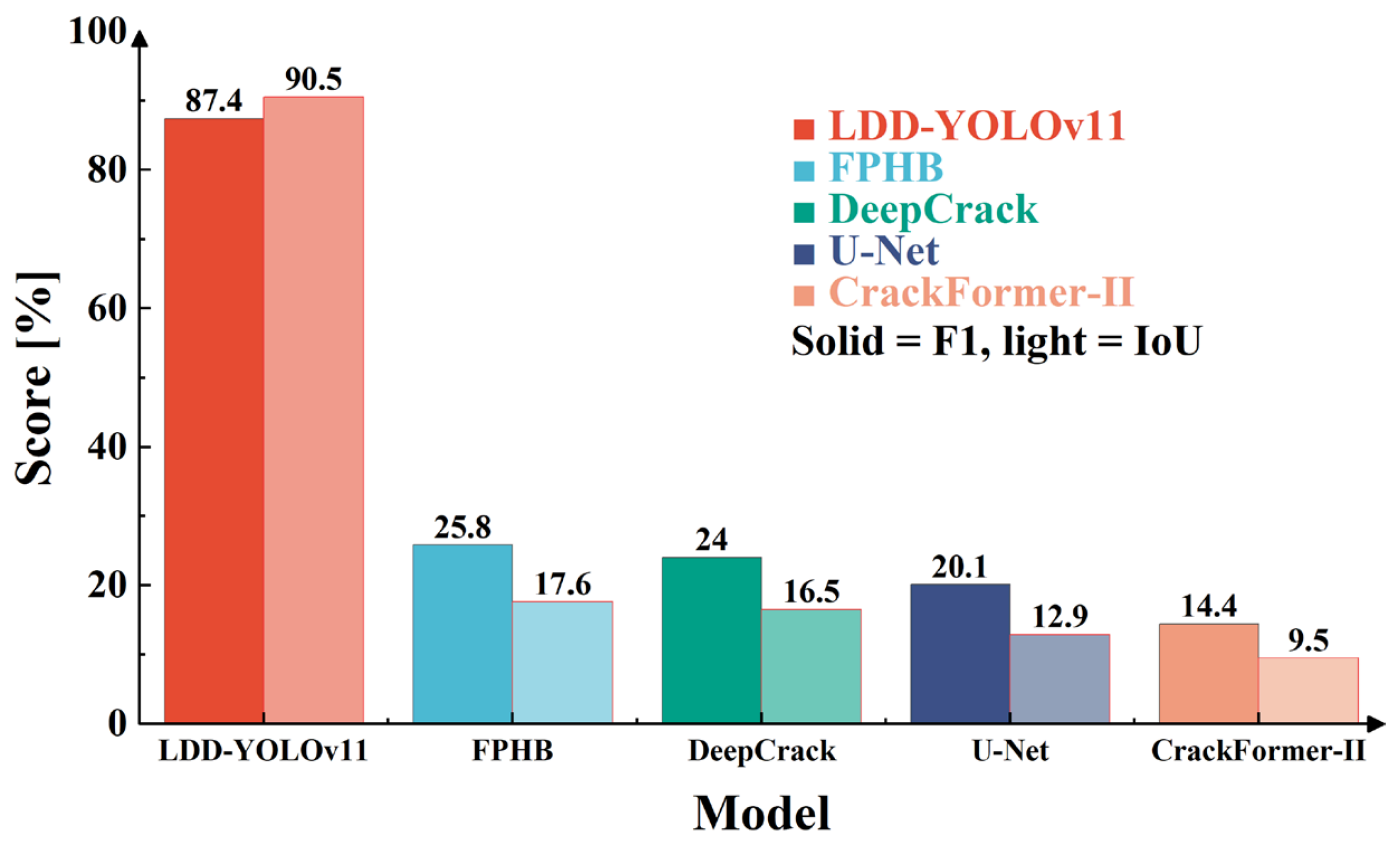
Box-level F1 score and IoU comparison with semantic segmentation models.

**Figure 17:**
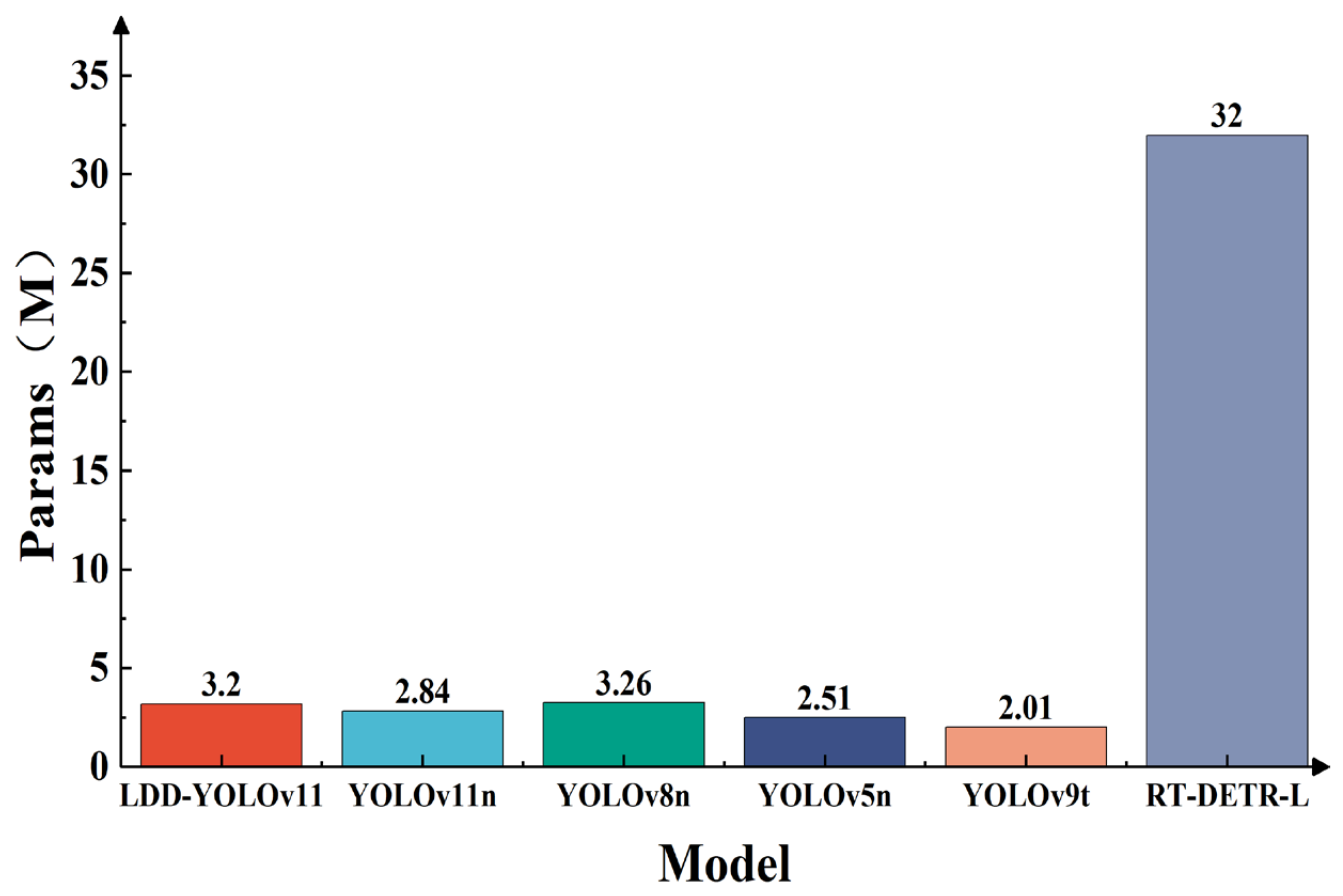
Parameter quantity (Params) comparison of different models.

**Figure 18:**
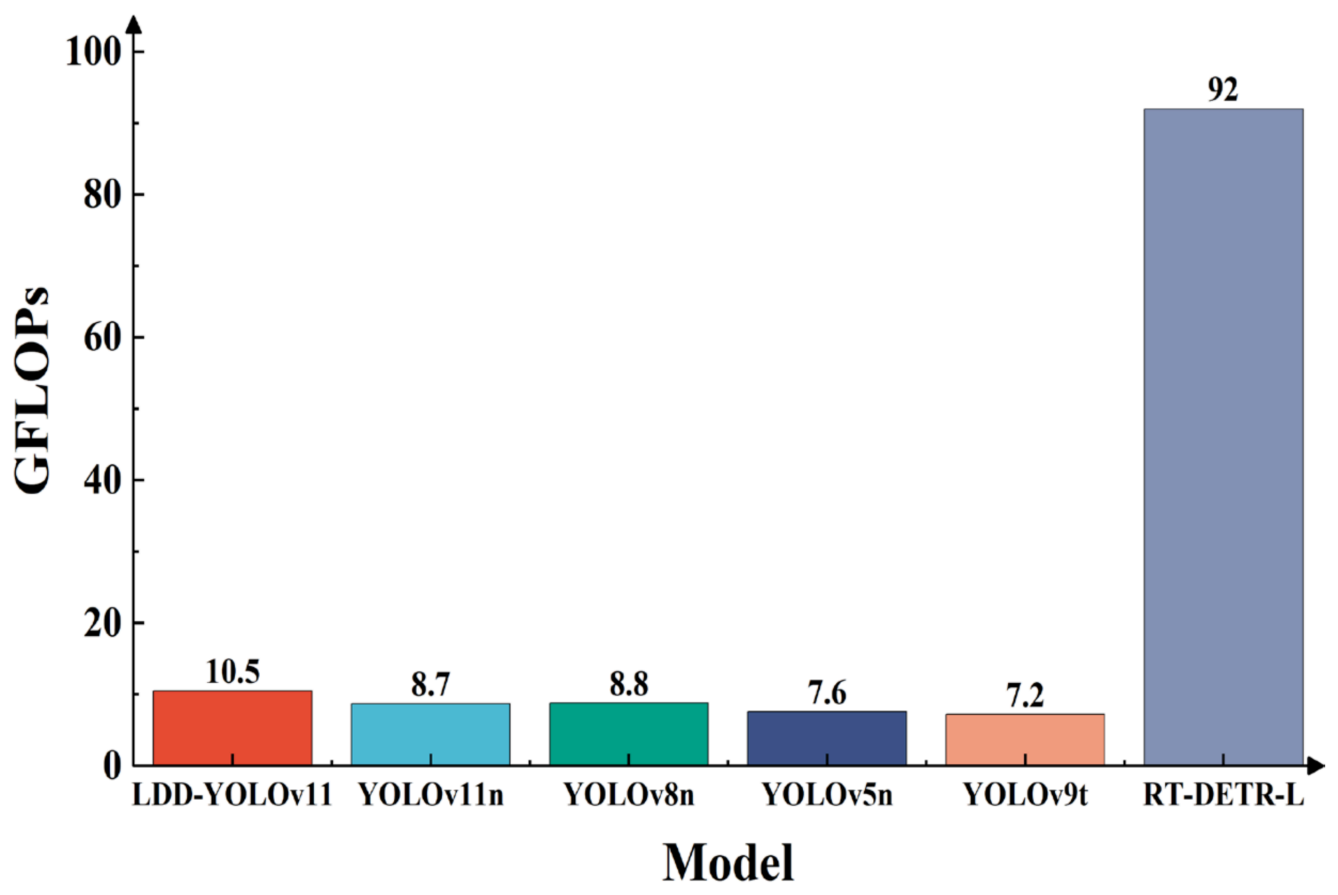
Computational complexity (GFLOPs) comparison of different models.

**Figure 19:**
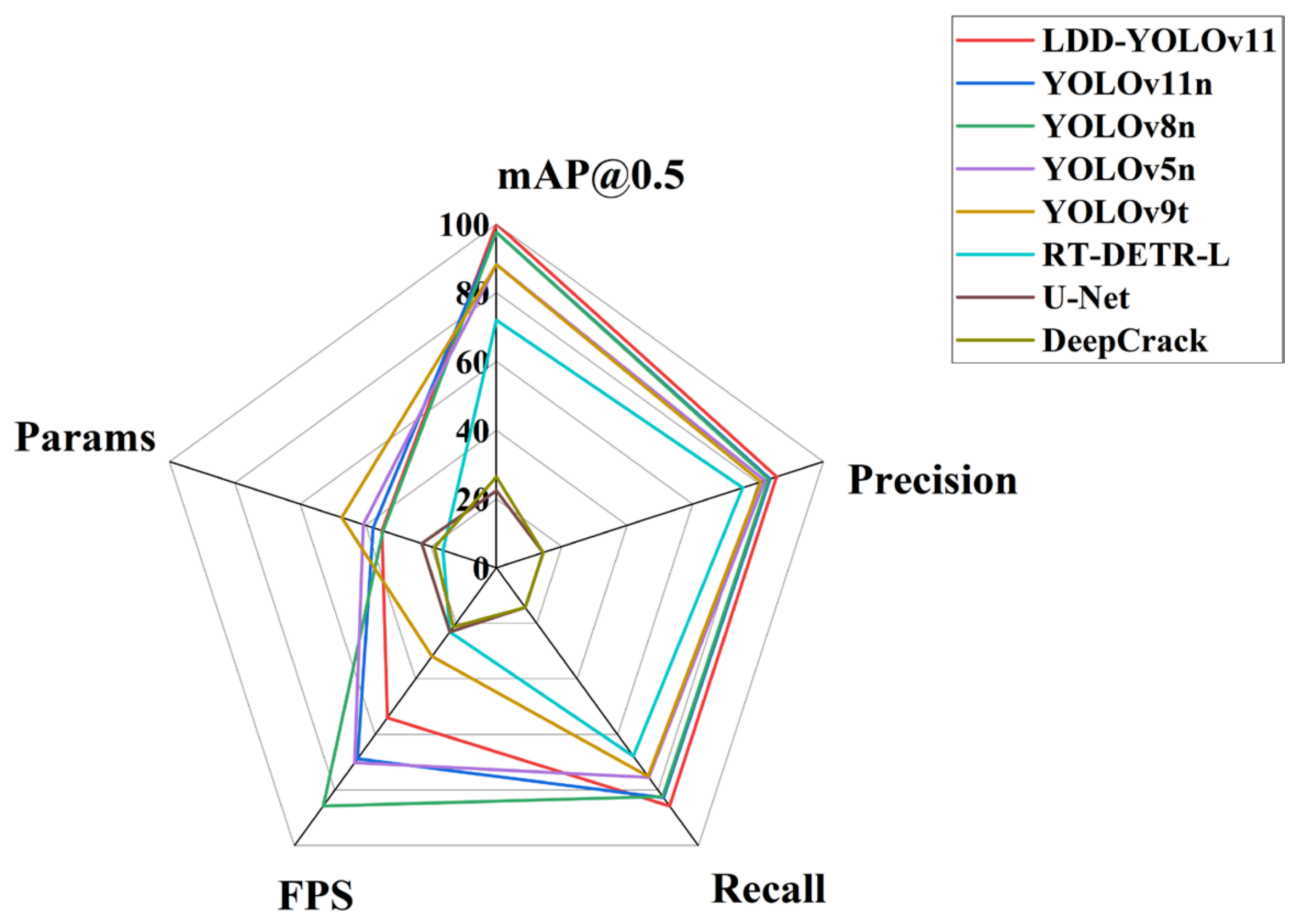
Performance radar chart for different models.

**Figure 20:**
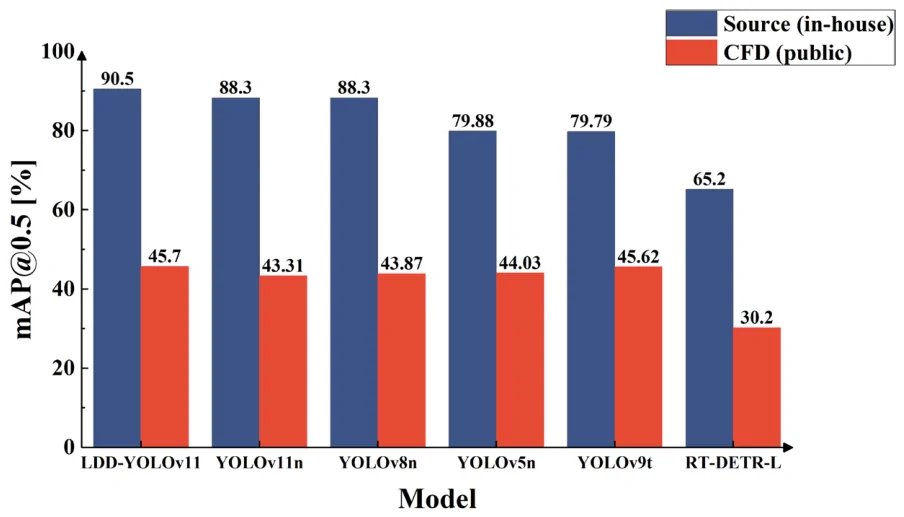
Cross-dataset generalization performance comparison.

**Figure 21:**
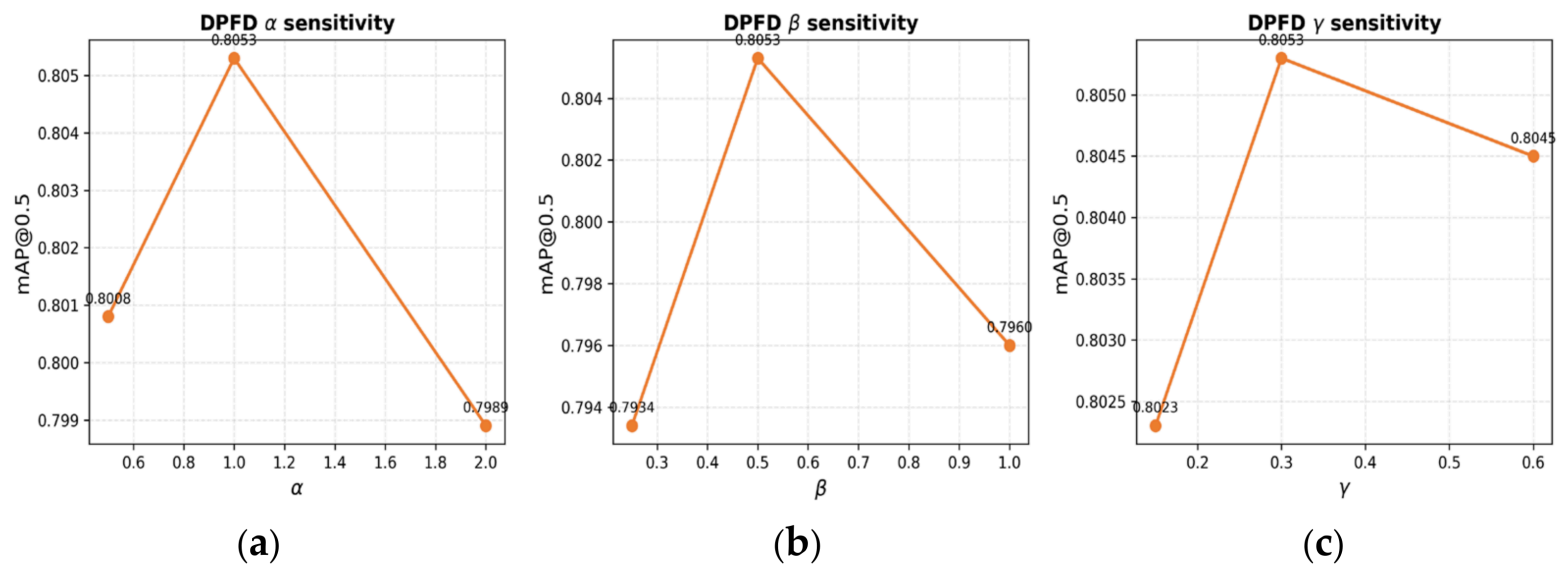
Sensitivity analysis of DPFD weight coefficients. (**a**) mAP@0.5 variation with feature distillation weight α; (**b**) mAP@0.5 variation with response distillation weight β; (**c**) mAP@0.5 variation with prototype contrast weight γ. All experiments are conducted under the condition that other hyperparameters remain fixed.

**Figure 22:**
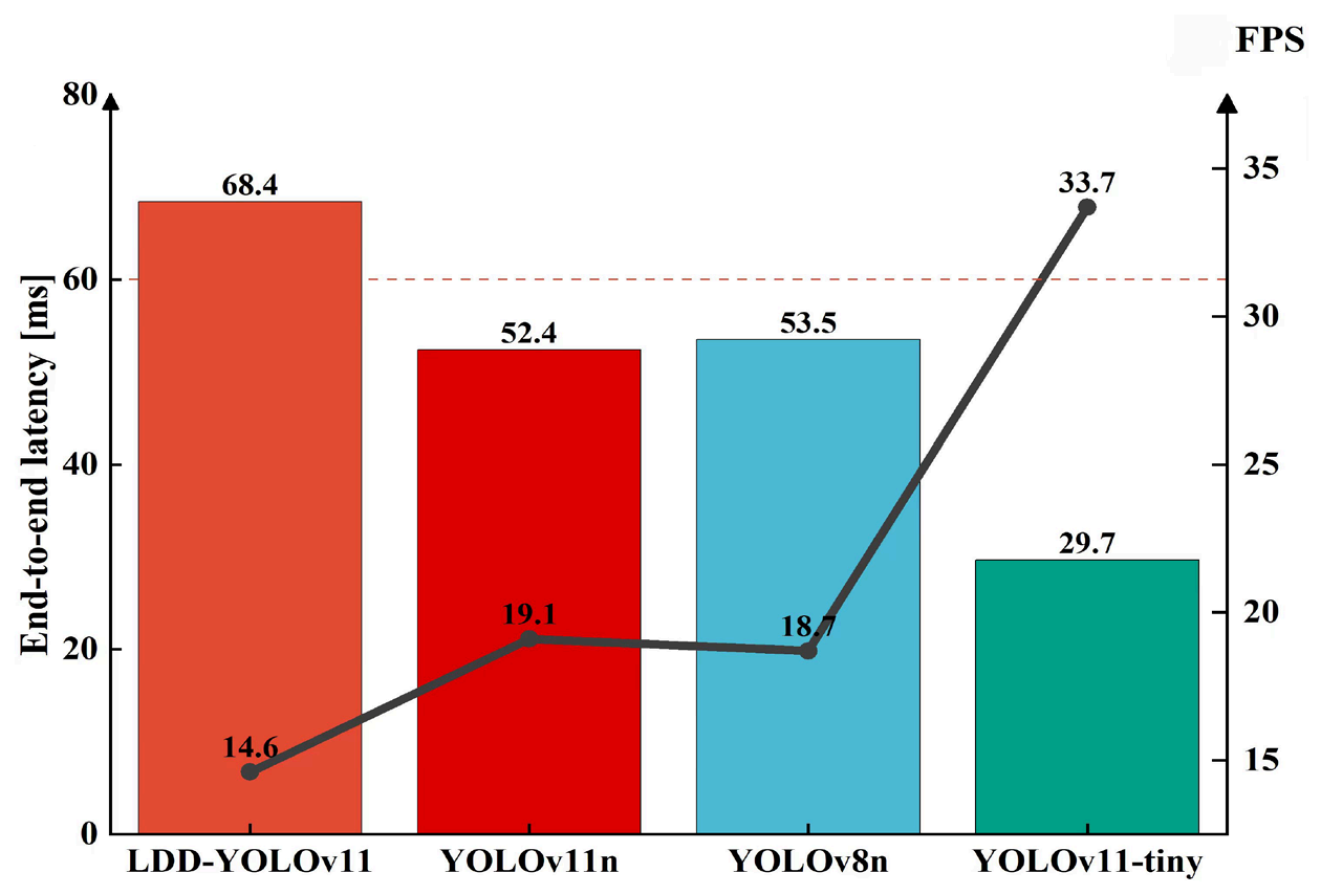
Inference performance comparison on general-purpose CPU hardware. The left-hand vertical axis is the end-to-end latency (ms), and the right-hand vertical axis is the frame rate (FPS). Bars represent single-frame end-to-end inference latency, and the line plot shows the corresponding inference FPS.

**Figure 23:**
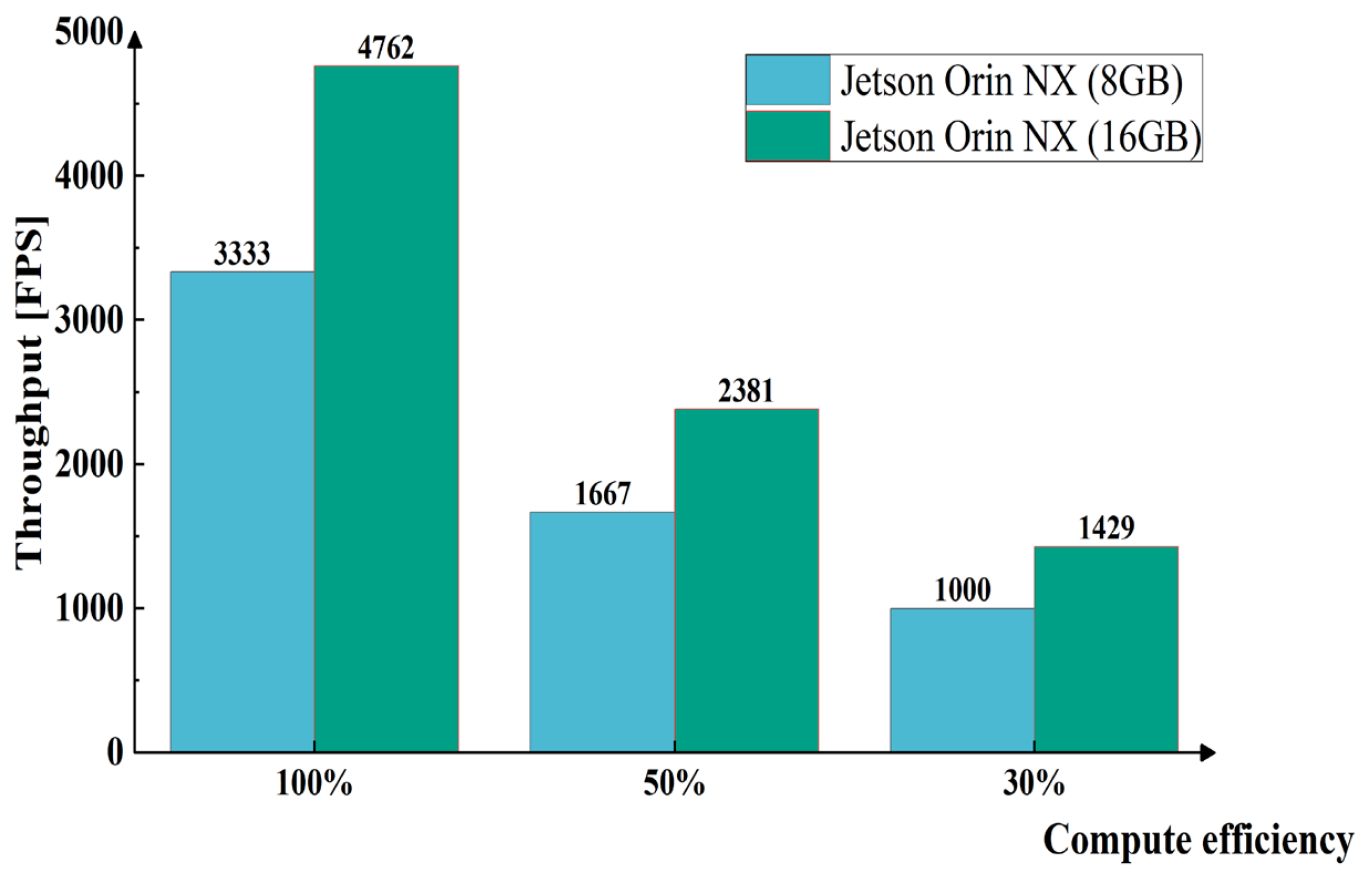
Simulated inference throughput on Jetson Orin NX under different computing-efficiency constraints.

**Table 1:** Detailed statistics of the self-built building crack dataset.

Category	Subcategory	Training Set	Test Set	Total
Basic information	Number of independent buildings	36	14	50
	Number of images	4012	988	5000
	Image resolution	640 × 640	640 × 640	-
Crack width level	Ultra-fine cracks (<1 mm)	1125	287	1412
	Medium cracks (1–5 mm)	2103	506	2609
	Wide cracks (>5 mm)	784	195	979
Illumination condition	Strong light	1284	312	1596
	Normal light	1876	465	2341
	Low light	852	211	1063
Background interference	Water stains	956	231	1187
	Masonry joints	1328	327	1655
	Stains/clutter	729	184	913

Note: The background-interference categories are not mutually exclusive; a single image may contain multiple interference types or none, so the sum of these counts is less than the total number of images.

**Table 2:** Summary of training hyperparameters.

Category	Parameter	Value/Setting
Base Training Configuration	Input image resolution	640 × 640
	Total training epochs	300
	Batch size	16
	Warm-up epochs	5
Optimizer Configuration	Optimizer type	SGD (Stochastic Gradient Descent)
	Initial learning rate	0.01
	Learning rate scheduler	Cosine annealing
	Momentum	0.937
	Weight decay	0.0005

**Table 3:** Comparison of detection model performance.

Method	P (%)	R (%)	FPS	Params (M)	mAP@0.5 (%)
LDD-YOLOv11	91.6	83.5	67.7	3.20	90.50
YOLOv11n	89.0	80.0	92.9	2.84	88.30
YOLOv8n	88.5	79.5	121.9	3.26	88.30
YOLOv5n	87.0	71.4	95.3	2.51	79.88
YOLOv9t	85.4	71.0	30.0	2.01	79.79
RT-DETR-L	78.3	62.5	15.0	32.00	65.20
U-Net	-	-	15.0	7.76	20.30
DeepCrack	-	-	12.0	14.20	23.90

## Data Availability

The CrackForest (CFD) dataset is available at https://github.com/cuilimeng/CrackForest-dataset, accessed on 21 October 2026. The Concrete Crack Images for Classification dataset (CCIC) is available at https://doi.org/10.17632/5y9wdsg2zt.2. The self-constructed building crack dataset and the source code that support the findings of this study are available from the corresponding author upon reasonable request.

## Abbreviations

The following abbreviations are used in this manuscript:

LMA	Light-Material Aware Adapter
DMC-Focus	Direction-Material Constrained Focus
DPFD	Dual Perception Focusing Distillation
